# Agmatine as the initiating substrate for aspartate semialdehyde-dependent bacterial spermidine biosynthesis

**DOI:** 10.1016/j.jbc.2026.113570

**Published:** 2026-09-17

**Authors:** Johnette C. Ostlund, Lindsey A. Johnston, Makenzie A. Pardoe, Isabel M. Bustillos, Megan L. Bizzell, Savannah J. Jones, Danielle F. Lee, Jeffrey S. McFarlane

**Affiliations:** Department of Chemistry and Biochemistry, Fort Lewis College, Durango, Colorado, USA

**Keywords:** bacteria, polyamine, spermidine, agmatine, putrescine, diaminopropane, carboxynorspermidine, carboxyspermidine, carboxyagminopropylagmatine, aminopropylagmatine

## Abstract

Putrescine has long been considered the initiating substrate for aspartate semialdehyde-dependent bacterial spermidine biosynthesis. However, two recent studies identified a variant pathway in which agmatine serves as the preferred substrate. This variation appears to be common, but the mechanistic basis for substrate preference was unknown. Here, we investigate the aspartate semialdehyde-dependent dehydrogenases from *Bacteroides fragilis* and *Clostridium leptum* using steady-state and transient-state kinetic assays, mass spectrometry, thermal shift protein stability assays, X-ray crystallography, small-angle X-ray scattering, and computational modeling. Both enzymes exhibit dramatically enhanced catalytic efficiency with agmatine, demonstrating that these homologs function as carboxyaminopropylagmatine dehydrogenases rather than carboxyspermidine dehydrogenases. Agmatine enhances NADPH binding affinity by 34-fold, whereas putrescine has a modest effect and at concentrations less likely to be physiologically relevant. Apo crystal structures and small angle X-ray scattering analysis reveal substantial ligand-dependent conformational rearrangements involving both global domain and active-site loop motions. These structural transitions, together with thermal stability measurements and transient-state binding assays, support an induced-fit mechanism in which agmatine promotes conformational rearrangements that enhance NADPH-binding. Comparative sequence analysis of homologs and ligand docking lead us to propose an active-site recognition loop governing discrimination between agmatine, putrescine, and diaminopropane substrates. Together, these findings redefine the substrate specificity of the *B. fragilis* and *C. leptum* enzymes, establish a mechanistic model for agmatine-dependent catalysis in alternative spermidine biosynthesis, and provide a testable framework for predicting substrate specificity across this family of bacterial polyamine biosynthetic enzymes.

Spermidine is an abundant polyamine in bacteria and is typically synthesized using putrescine as a precursor (1). Many bacterial species generate putrescine through decarboxylation of arginine to form agmatine, followed by ureohydrolysis of agmatine to generate putrescine. Two distinct bacterial pathways have been described that utilize putrescine in spermidine formation. In the S-adenosyl-L-methionine-dependent pathway, spermidine synthase transfers an aminopropyl group from decarboxylated S-adenosyl-L-methionine to putrescine, forming spermidine (2) (Fig. 1*A*). The aspartate semialdehyde (ASA)-dependent pathway, described over 3 decades ago, utilizes ASA to produce spermidine through a coupled enzyme reaction involving carboxyspermidine dehydrogenase (CASDH) and carboxyspermidine (CSP) decarboxylase (3, 4, 5). CASDH catalyzes an NADPH-dependent condensation of ASA with putrescine to form CSP, which is decarboxylated by CSP decarboxylase to produce spermidine (Fig. 1*B*). Two prevalent gut microbes, *Bacteroides fragilis* and *Clostridium leptum*, use the ASA dependent pathway, which is encoded in the genomes of 70% of human gut species (6). Previous kinetic studies of CASDH from these species demonstrated activity with both putrescine and diaminopropane (which replaces putrescine in a variation of the alternative pathway forming norspermidine as the final product). However, the reported mM *k*_*m*_ values for putrescine and diaminopropane indicated poor catalytic efficiency (7).

**Figure 1:**
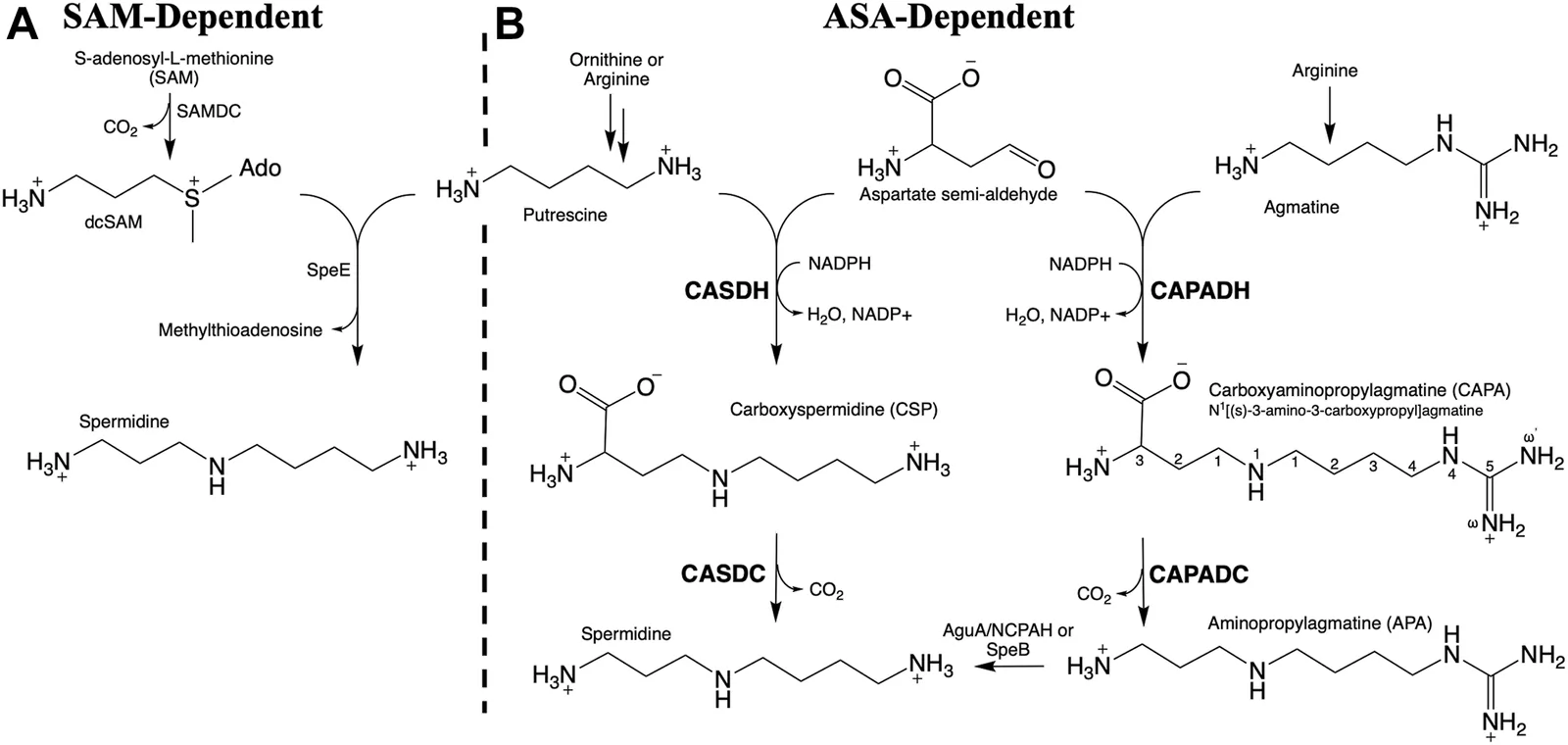
**Comparison of bacterial spermidine biosynthetic pathways.***A*, S-adenosyl-L-methionine (SAM) dependent pathway. *B*, aspartate semialdehyde (ASA) dependent pathway. AguA, deiminase; NCPAH, amidohydrolase; SpeB, ureohydrolase; CAPADH, carboxyaminopropylagmatine dehydrogenase; CASDH, carboxyspermidine dehydrogenase; CAPADC, carboxyaminopropylagmatine decarboxylase; CASDC, carboxyspermidine decarboxylase; SpeE, spermidine synthase; SAMDC, SAM decarboxylase.

Recently, two independent studies identified several CASDH homologs that preferentially utilize agmatine rather than putrescine as substrates (8, 9). These data suggest that subclasses of ASA-dependent dehydrogenases exist, with divergent substrate specificity. Xi *et al.* used mass spectrometry assays of cell extracts to demonstrate that the cyanobacterium *Synechocystis* sp. PCC 6803 produces spermidine through a previously unrecognized intermediate, carboxyaminopropylagmatine (CAPA). Instead of CASDH, *Synechocystis* encodes a carboxyaminopropylagmatine dehydrogenase (CAPADH), which catalyzes the reductive condensation of ASA and agmatine to produce CAPA. CAPA is decarboxylated by carboxyaminopropylagmatine decarboxylase (CAPADC) to produce aminopropylagmatine (APA) as a precursor to spermidine, establishing a new variation of the ASA dependent biosynthetic pathway (Fig. 1) (9). Importantly, the *Synechocystis* CAPADH was a poor catalyst with putrescine or diaminopropane as substrates, with mM *k*_*m*_ values. Li *et al.* expanded the examination of agmatine utilization by constructing heterologous expression systems and using mass spectrometry assays to demonstrate the existence of CAPADH/CAPADC dependent pathways in diverse species such as *Desulfovibrio desulfuricans*, *Hydrogenimonas thermophila,* and in *C. leptum* (8).

Despite the discovery of agmatine-dependent spermidine biosynthesis, it was unclear how ASA-dependent dehydrogenases utilizing agmatine might be distinguished from homologs that preferentially select putrescine or diaminopropane. Here, we report steady-state, mass spectrometry and transient-state stopped-flow assays to demonstrate that agmatine is the initiating substrate for spermidine biosynthesis in *B. fragilis* and to corroborate the findings of Li *et al.* in *C. leptum*. We observed that NADPH binding was enhanced in the presence of agmatine. A combination of thermal shift protein stability assays, transient-state binding assays, X-ray crystallography, small angle X-ray scattering (SAXS), and comparative sequence and structural analysis were used to develop a model describing agmatine’s promotion of productive NADPH binding and catalysis, and to identify structural determinants that distinguish agmatine-, putrescine-, and diaminopropane-dependent members of this enzyme family.

## Results

### Coupled mass spectrometry assay of spermidine production

A coupled enzyme assay using mass spectrometry was originally developed to test the production of spermidine by the *B. fragilis* dehydrogenase and decarboxylase enzymes using putrescine as a substrate. This was of particular interest because these enzymes are located on separate operons that are not directly associated with polyamine production (rather, they encode genes associated with DNA repair). We heterologously expressed and purified the *B. fragilis* dehydrogenase and decarboxylase enzymes using nickel-chelating Sepharose affinity chromatography as described in this article and previously (7). The dehydrogenase (4 μM) was allowed to react for 1 h at 25 °C with 200 μM NADPH, 2 mM ASA and 20 mM putrescine in 25 mM Tris pH 7.5 buffer. The decarboxylase (1 μM) was added, and the reaction was incubated for an additional hour prior to quenching, addition of ^13^C spermidine (spermidine (butyl-^13^C4) trihydrochloride) as an internal standard, isobutyl chloroformate derivatization, extraction of the derivatized spermidine product and analysis by LC-MS/MS (Fig. 2 and Table S1). Spermidine was clearly produced, but only 9 μM which was surprisingly little. This indicated low catalytic activity by the decarboxylase with CSP as a substrate. While considering this puzzle, we noted in papers by Xi (9) and Li (8) that agmatine had been demonstrated to be the preferred substrate in some species and we received a personal communication from Dr Anthony Michael encouraging us to investigate agmatine as a substrate for *C. leptum*.

**Figure 2:**
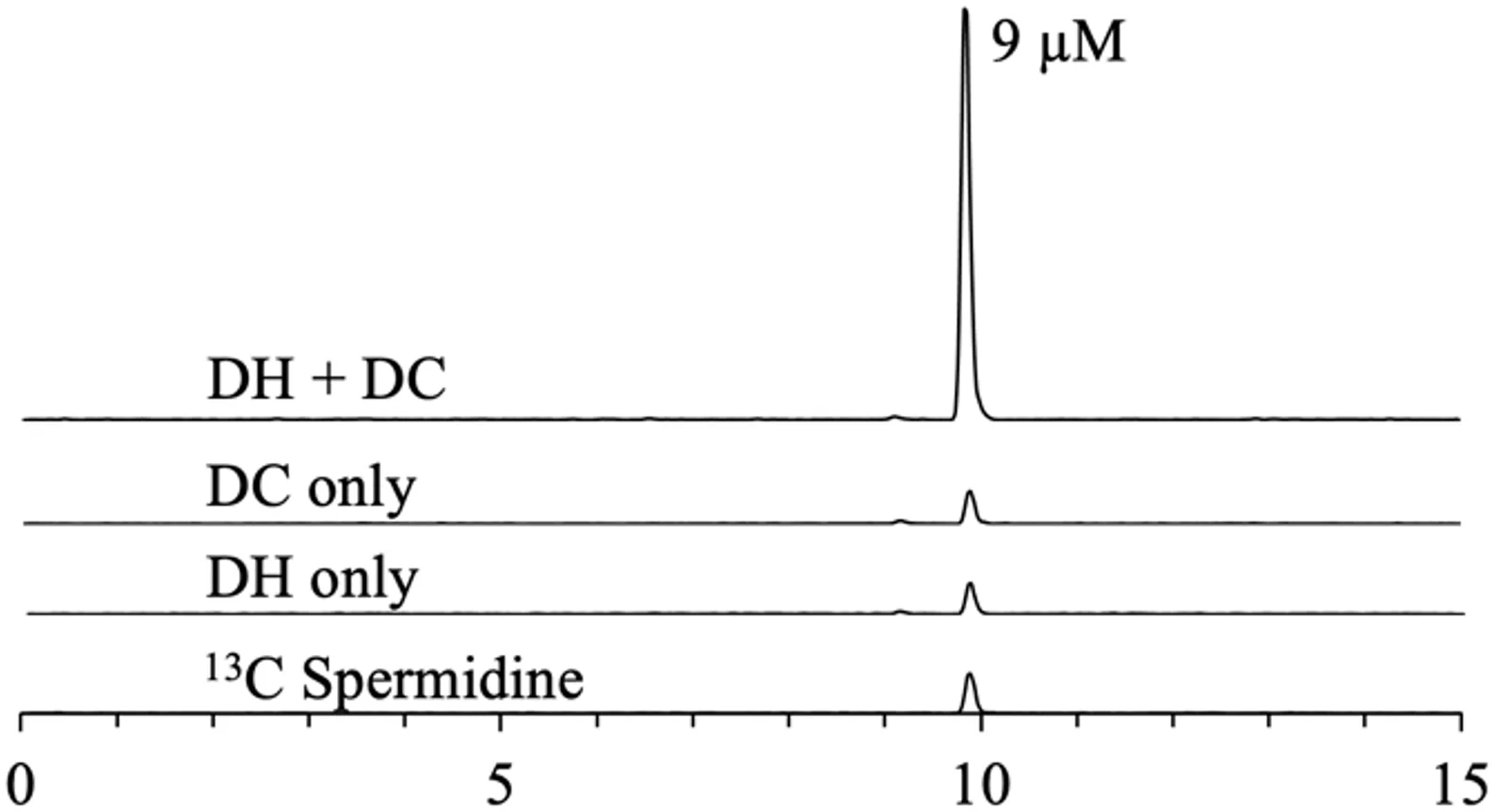
***Bacteroides fragilis* LC-MS/MS assay of spermidine biosynthesis.** Extracted ion chromatograms of the 372 *m/z* [M + H]^+^, isobutyl chloroformate derivatized, spermidine product ion. DH + DC coupled assay. BfCAPADH at 4 μM was reacted with 200 μM NADPH, 2 mM aspartate-semialdehyde, and 20 mM putrescine and monitored to completion at 25 °C to produce carboxyspermidine (CSP). BfCAPADC at 1 μM was added and allowed to react with CSP for 1 h at 25 °C. Reaction was quenched, spiked with ^13^C spermidine as an internal standard, derivatized and the resulting spermidine precursor was selected at 446 *m/z* [M + H]^+^ prior to collision induced dissociation and product ion detection at 372 *m/z* [M + H]^+^. DC only and DH only control reactions were conducted in the absence of one enzyme. ^13^C spermidine contains a trace amount of ^12^C spermidine resulting in a small spermidine peak in each sample. Each experiment was repeated three times. Traces shown are a representative experiment.

### Steady-state kinetic assays of CAPADH with agmatine as a substrate

We performed steady-state kinetic measurements for the dehydrogenase enzymes from *B. fragilis* and *C. leptum* using agmatine as the initiating substrate. In these assays, the oxidation of NADPH was monitored by observing loss of absorbance at 340 nm as the imine intermediate was reduced to the final product, CAPA. Both enzymes were dramatically more efficient when using agmatine than they were with putrescine or diaminopropane (Table 1, Figs. 3 and S1). Indeed, the *k*_*cat*_/*K*_*m*_ increased 115-fold for *B. fragilis* and 811-fold for *C. leptum* in comparison to putrescine. The *K*_*m*_ values for agmatine were 172 μM and 63 μM for *B. fragilis* and *C. leptum* respectively, a much more plausible physiologic concentration than the 4.2 mM and 5.0 mM *k*_*m*_ values previously measured for putrescine. These results led us to compare CAPA and CSP as substrates for the *Bacteroides fragilis* decarboxylase (BfCAPADC).

**Table 1:** CAPADH steady state kinetics

Enzyme	Substrate	*k*_*cat-app*_*(s*^*−1*^*)*	*K*_*m*_	*k*_*cat*_*/K*_*m*_*(M*^*−1*^*s*^*−1*^*)*
*Bf*CAPADH^a^	Putrescine	0.41 ± 0.02	4.2 ± 0.3 mM	96 ± 7
With Putrescine	ASA	0.64 ± 0.02	2.7 ± 0.1 mM	230 ± 15
	NADPH	0.39 ± 0.05	57 ± 0.3 μM	6700 ± 200
*Bf*CAPADH	Agmatine	1.9 ± 0.1	172 ± 22 μM	11,000 ± 2000
with Agmatine	ASA	2.4 ± 0.1	68 ± 3 μM	36,000 ± 2000
	NADPH	3.6 ± 0.3	1.6 ± 0.3 μM	2.3 × 10^6^± 5 × 10^5^

a Previously published (7).

**Figure 3:**
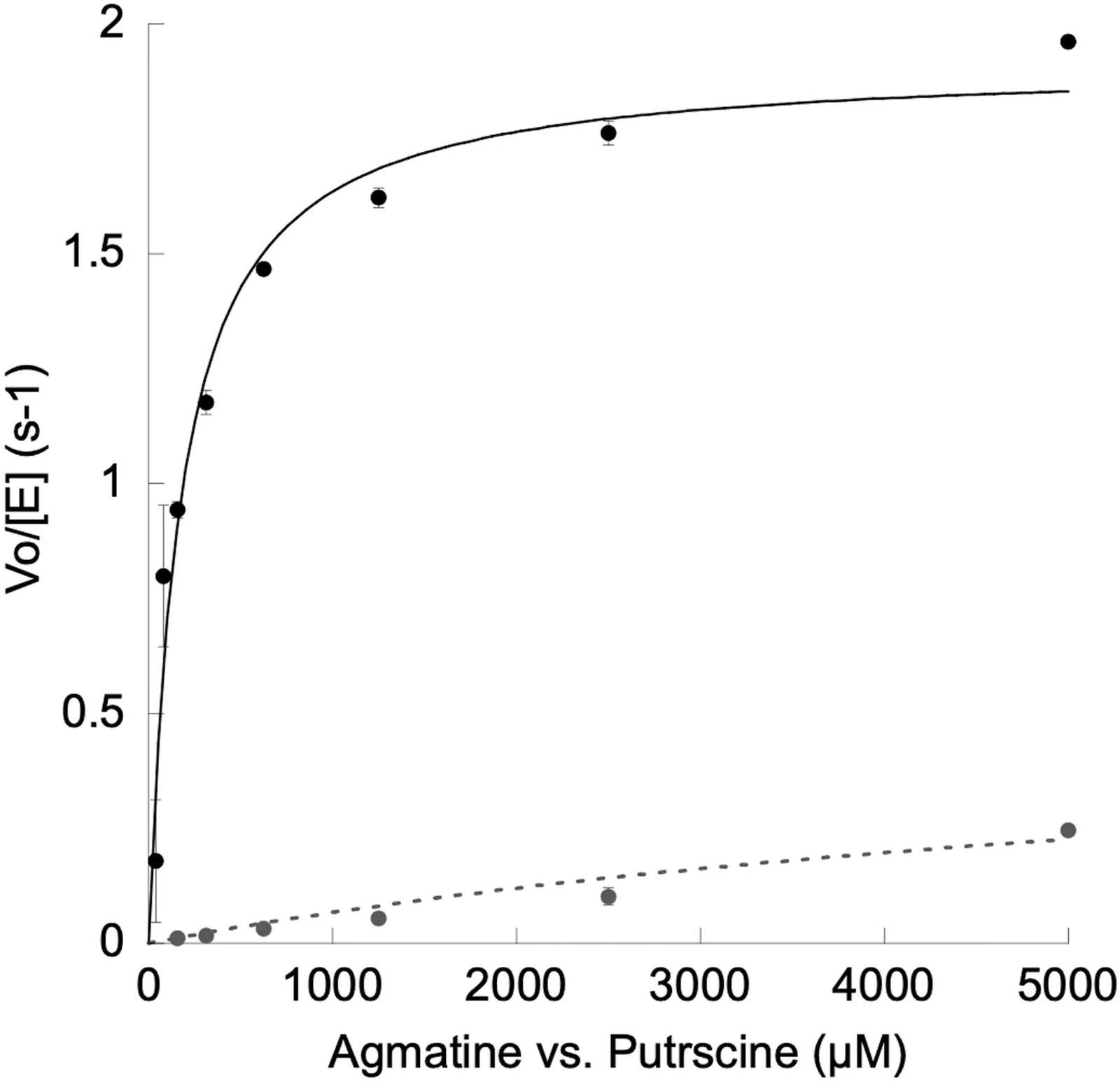
**Steady-state kinetic comparison of agmatine and putrescine for BfCAPADH.** Initial rates were determined by applying linear fits to primary plots of NAD(P)H oxidation at 340 nm. Secondary plots were fit to the Michaelis-Menten equation. Agmatine reaction (*solid line*) - 1 μM BfCAPADH, 1 mM ASA (aspartate semialdehyde), 150 μM NADPH, and varied agmatine (78 μM - 5 mM). Putrescine reaction (*dashed line*) - 1 μM BfCAPADH, 2.5 mM ASA, 150 μM NADPH, and varied putrescine (156 μM – 5 mM). Error bars represent the SD of at least three trials. ASA, aspartate semialdehyde.

### Coupled stopped-flow assays of APA production

Derivatizing the guanidinium group of CAPA using isobutyl chloroformate was not successful, likely due to resonance stabilization of that functional group. Accordingly, a coupled stopped-flow spectrometry assay was employed to measure conversion of CAPA to APA. BfCAPADC is pyridoxal-5′-phosphate (PLP)-dependent, and this assay was predicated on observing the PLP spectral changes that occur during a decarboxylation catalytic cycle (10). Purified BfCAPADC had an absorbance peak at 420 nm indicating that PLP was bound in the active site, but supplementation of PLP increased absorbance by ∼30% as described in methods. The reaction was conducted at 4 °C to slow and capture rapid spectral transitions. BfCAPADH was reacted with 1.25 mM agmatine, 1 mM ASA, and 150 μM NADPH to generate CAPA. The oxidation of NADPH was monitored at 340 nm to determine the amount of CAPA produced (typically 95% of the NADPH was consumed). BfCAPADC (25 μM) was rapidly mixed with BfCAPADH reactions containing 50 – 6.25 μM CAPA and monitored on a TgK stopped-flow spectrometer equipped with a KinetaScan CCD by scanning from 305 to 550 nm every 1.6 ms for 3.2 s. All concentrations described are the final, mixed concentration in the stopped-flow cell. The spectral changes observed had a fast phase with a rapid gain of signal at 335 nm and loss of signal at 420 nm, and a slower phase with equivalent signal trends. We interpreted these changes as representing the end of the catalytic cycle, likely the release of APA from the gem diamine and the return to and equilibration of the ketoenamine and enolimine forms of PLP internal aldimine. These data were analyzed in Kintek Explorer (11) by single value decomposition and fit to a two-step irreversible model (12) (Figs. 4 and S2). This analysis gave a fast-phase rate constant *k*_*1*_ = 15 ± 3 s^−1^ and a slow-phase rate constant of *k*_*2*_ = 1.5 ± 0.3 s^−1^. The experiment was conducted twice and the rate constants for separate experiments were within error. In the absence of CAPA, no change in PLP signal was observed. The experiment was repeated using CSP. BfCAPADC (25 μM) was rapidly mixed with BfCAPADH reactions containing 50 – 6.25 μM CSP and monitored as described. The absorbance spectra showed very little change, even over time scales up to 314 s (Fig. S2). Capturing changes in PLP absorbance requires pre-steady state conditions, such that spectral shifts are observed as concerted changes among enzyme molecules in solution. We interpret the lack of signal change for CSP decarboxylation to suggest inefficient binding of CSP resulting in enzyme molecules and their respective PLP cofactors diverging to a variety of spectral states that negate any concerted signal change. These data suggest that the BfCAPADC preferentially selects the CAPA substrate, even though it will slowly utilize CSP as observed by mass spectrometry.

**Figure 4:**
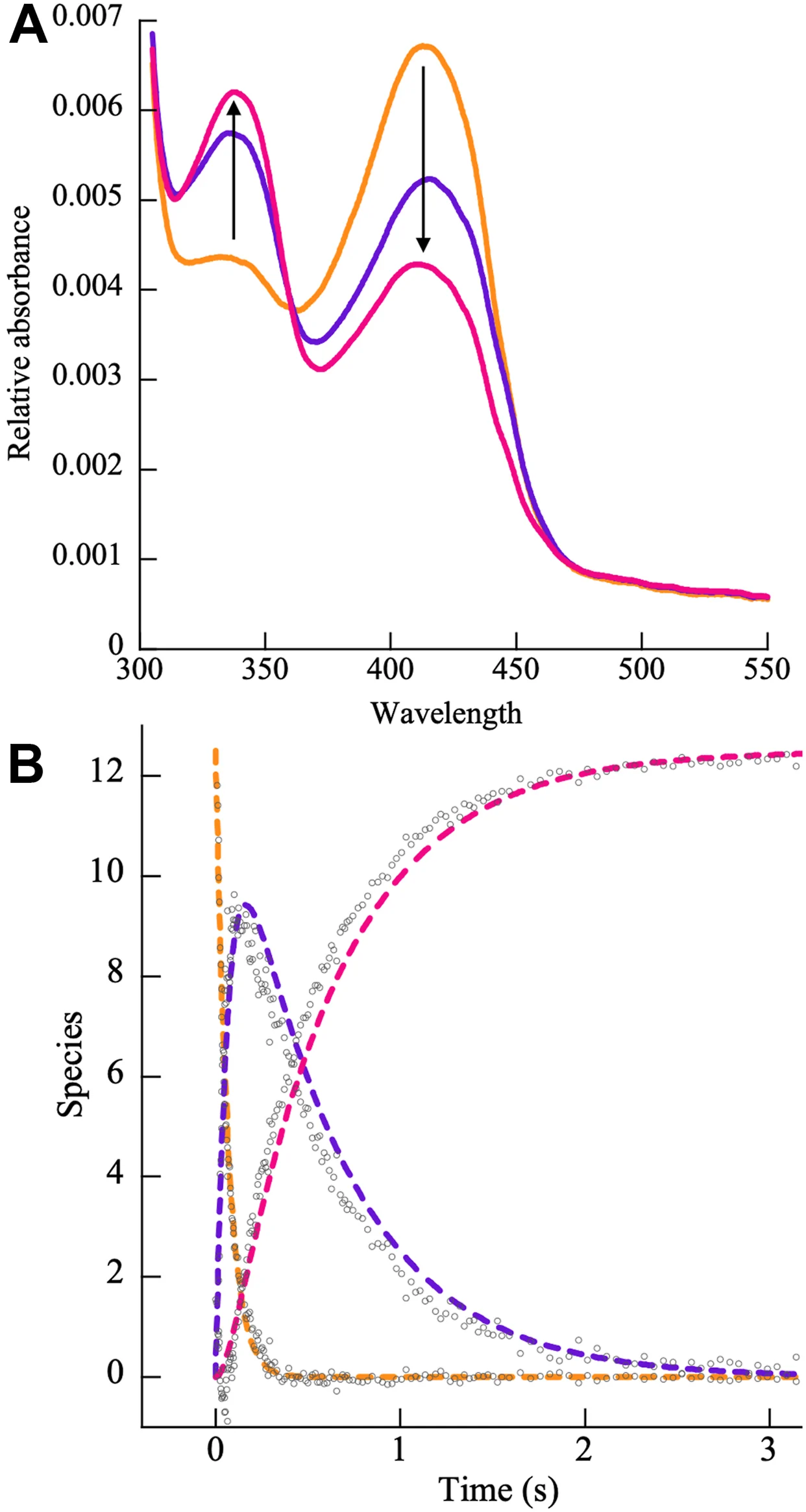
***Bacteroides fragilis* stopped-flow assay of aminopropylagmatine (APA) production.** Pyridoxal-5′-phosphate absorbance was monitored in log mode for 3.2 s by scanning from 305 to 550 nm using a TgK Scientific KinetaScan CCD with 1.6 ms integration time. All concentrations are the final concentration in the stopped-flow cell. BfCAPADC at 25 μM was rapidly mixed with 6.25 to 50 μM CAPA. Multiwavelength data were analyzed in Kintek Explorer by single value decomposition. *A*, reconstructed difference spectra from single value decomposition analysis. *Orange* – initial scan. *Pink* – final scan. *Purple* – intermediate scan. *B*, the number of intermediate species were estimated from data in *panel A* and data for the concentration series were globally fit to a two-step, irreversible model. The fits are represented as *dashed lines*. Data shown is a 12.5 μM CAPA trial and is representative of two experiments conducted on different days. Global fitting and fitspace data are found in Figure S2. BfCAPADC, *Bacteroides fragilis* decarboxylase; CAPA, carboxyaminopropylagmatine.

Taken together, our steady-state and transient-state data suggest that agmatine is the preferred substrate for *B. fragilis* and *C. leptum*. Thus, both *B. fragilis* and *C. leptum* encode CAPADH and not CASDH. *C. leptum* encodes a ureohydrolase (putative SpeB ureohydrolase - Uniprot A0A855A6N2) that is predicted to remove urea from APA generating spermidine (Fig. 1). In contrast, *B. fragilis* is predicted to convert APA to spermidine through a two-step path using a deiminase (putative AguA deiminase - Uniprot Q5LCJ9) and an amidohydrolase (putative C-N hydrolase – Uniprot Q5LCK0). The deiminase removes NH_4_ from the guanidinium converting it to a carbamoyl group prior to removal by the amidohydrolase which generates spermidine. We considered the possibility that the deiminase could act on agmatine and generate carbamoylputrescine as a substrate for BfCAPADH. Accordingly, 1 mM and 125 μM carbamoylputrescine was tested as a substrate for BfCAPADH. The initial rate at 1 mM carbamoylputrescine was 68-fold lower than for agmatine and at 125 μM was within the limit of detection of our assay (<0.008 V_o_/[E] s^−1^) suggesting that carbamoylputrescine is not a viable substrate for BfCAPADH. Li *et al.* reported the use of spermidine as a substrate by CASDH enzymes from *Agrobacterium tumefaciens* and *Paracoccus denitrificans* generating carboxyspermine as a precursor to spermine. We tested 10 μM, 100 μM and 1 mM spermidine as a substrate for Bf- and Clostridium leptum carboxyaminopropylagmatine dehydrogenase (ClCAPADH) in our NADPH oxidation assay but observed maximal activity at 1 mM of only 0.017 s^−1^ for BfCAPADH (112-fold slower than agmatine *k*_*cat*_) and 0.13 s^−1^ for ClCAPDH (28-fold slower than agmatine *k*_*cat*_) suggesting that spermine production is not a major product.

Thus, *B. fragilis* and *C. leptum* encode CAPADH and CAPADC enzymes to produce APA which is expected to be converted to spermidine by a one- or two-step process converting the guanidinium group of APA to a primary amine. These results raised new questions about CAPADH catalysis. How is substrate specificity determined and what affect does agmatine have on the catalytic cycle?

### Thermal shift assays of CAPADH stability

CAPADH stability was first examined to explore potential crystallization conditions. Both Bf- and ClCAPADH had apo melting temperatures under 45 °C which is correlated with a decreased likelihood of crystallization (13). This led us to explore thermal stability of CAPADH in the presence of known ligands. Thermal stability was measured using a Sypro Orange thermal shift assay method wherein the fluorescence emission of the dye increases as it is stabilized in association with the non-polar core residues of an unfolding protein. CAPADH at 10 μM was combined with Sypro Orange and ligand at a concentrations 10-fold greater than the measured *K*_*m*_ (a concentration expected to be saturating). Increases in melting temperature as high as 7.7 °C for BfCAPADH and 12.3 °C for ClCAPADH were observed (Tables 2, S2, and Fig. S3).

**Table 2:** BfCAPADH thermal shift assay

Ligand	Bf - T_m_ (°C)	Cl - T_m_ (°C)
Apo	44.2 ± 0.4	44.9 ± 0.5
NADPH	44.3 ± 0.2	48.2 ± 0.6
Agmatine	44.3 ± 0.2	45.4 ± 0.3
Agmatine and NADPH	51.9 ± 0.7	57.2 ± 2.6
Putrescine	44.9 ± 0.2	44.7 ± 0.6
Putrescine and NADPH	45.3 ± 0.3	48.1 ± 0.2
ASA	47.0 ± 0.3	48.0 ± 0.3
ASA and NADPH	46.8 ± 0.6	51.0 ± 0.8
NADP+	48.4 ± 0.1	51.6 ± 0.2
Agmatine and NADP+	48.9 ± 0.6	53.0 ± 0.7

BfCAPADH (1 μM) was incubated with ligand at concentrations 10-fold above the measured *K*_m_ values prior to temperature-dependent denaturation in an RT-PCR instrument monitored as a fluorescence emission change with a Sypro Orange probe.

Agmatine had a surprising effect on stability. The *T*_*m*_ for Apo BfCAPADH was 44.2 ± 0.4 °C. With NADPH bound, the value was unchanged (+0.1 ± 0.2 °C). Likewise, agmatine alone did not alter the *T*_*m*_ (+0.1 ± 0.2 °C). Interestingly, when agmatine and NADPH were combined a synergistic effect was observed and the *T*_*m*_ increased to the largest value measured, +7.7 °C to 51.9 ± 0.7 °C. The presence of putrescine and NADPH had only a negligible effect (+1.1 ± 0.3 °C). ClCAPADH followed a similar pattern. NADPH alone was stabilizing (+3.3 ± 0.6 °C) while agmatine alone (+0.5 ± 0.3 °C) and putrescine alone (−0.2 ± 0.6 °C) had minimal effect, but when agmatine and NADPH were combined the *T*_*m*_ increased by 12.3 °C to 57.2 ± 2.6 °C. As with BfCAPADH, the combination of putrescine and NADPH caused no increase over NADPH alone (−0.1 ± 0.2 °C). These data suggested that binding of agmatine and NADPH by CAPADH might be interdependent.

Several other observations were noteworthy. ClCAPADH had a broader range of *T*_*m*_ values than BfCAPADH and was stabilized by NADPH alone suggesting potential conformational differences between the homologs. ASA had a modest stabilizing effect on both enzymes (Bf +2.8 ± 0.3 °C; Cl +3.1 ± 0.3 °C), but ASA and agmatine combined did not increase *T*_*m*_ further. We had observed that NADP^+^ improved crystallization for BfCAPADH and found that NADP^+^ increased *T*_*m*_ by 4.2 ± 0.1 °C for BfCAPADH and 6.7 ± 0.2 °C for ClCAPADH.

### NADPH binding assays

The nearest homolog to ASA-dependent dehydrogenase is saccharopine dehydrogenase (7). Although the active site arrangement differs significantly, saccharopine dehydrogenase from yeast catalyzes the reductive condensation of L-glutamate and L-α-aminoadipate semialdehyde to form saccharopine. This enzyme has been shown to follow an ordered mechanism where NADH binds, followed by the semialdehyde and finally L-glutamate (14). Saccharopine is released first, and NAD^+^ is released last. Thus, we hypothesized that CAPADH would bind NADPH first, followed by ASA and finally agmatine. To test this, we developed an NADPH binding assay.

The NADPH nicotinamide ring is fluorescent with an excitation λ_max_ at 340 nm and an emission λ_max_ at 450 nm. Capitalizing on this feature, BfCAPADH binding of NADPH was monitored by measuring total fluorescence change beyond a 360 nm cut-off filter, following excitation at 340 nm, with a TgK Scientific rapid-mixing, stopped-flow spectrometer. All concentrations described are the final, mixed concentration in the stopped-flow cell. The NADPH emission signal was observed to increase when combined with BfCAPADH. NADPH is known to adopt self-quenching conformational states in solution (15) and we interpreted the increase in emission to represent the stabilization of the NADPH in an extended conformation, when bound in the active site, as observed for other dehydrogenases (16). In a series of five experiments, as described in methods, 15 μM BfCAPADH was rapidly mixed with NADPH varied from 0.47 μM to 60 μM. Data were globally fit to a one-step equilibrium binding model 1 in Kintek Explorer (11) (Figs. 5*A* and S4). The resulting FitSpace (12) was well constrained. The calculated parameters for NADPH binding were *K*_*NADPH*_ = 61 μM, *k*_*1*_ = 1.0 μM^−1^s^−1^ and *k*_*-1*_ = 61 s^−1^ (Table 3).

**Figure 5:**
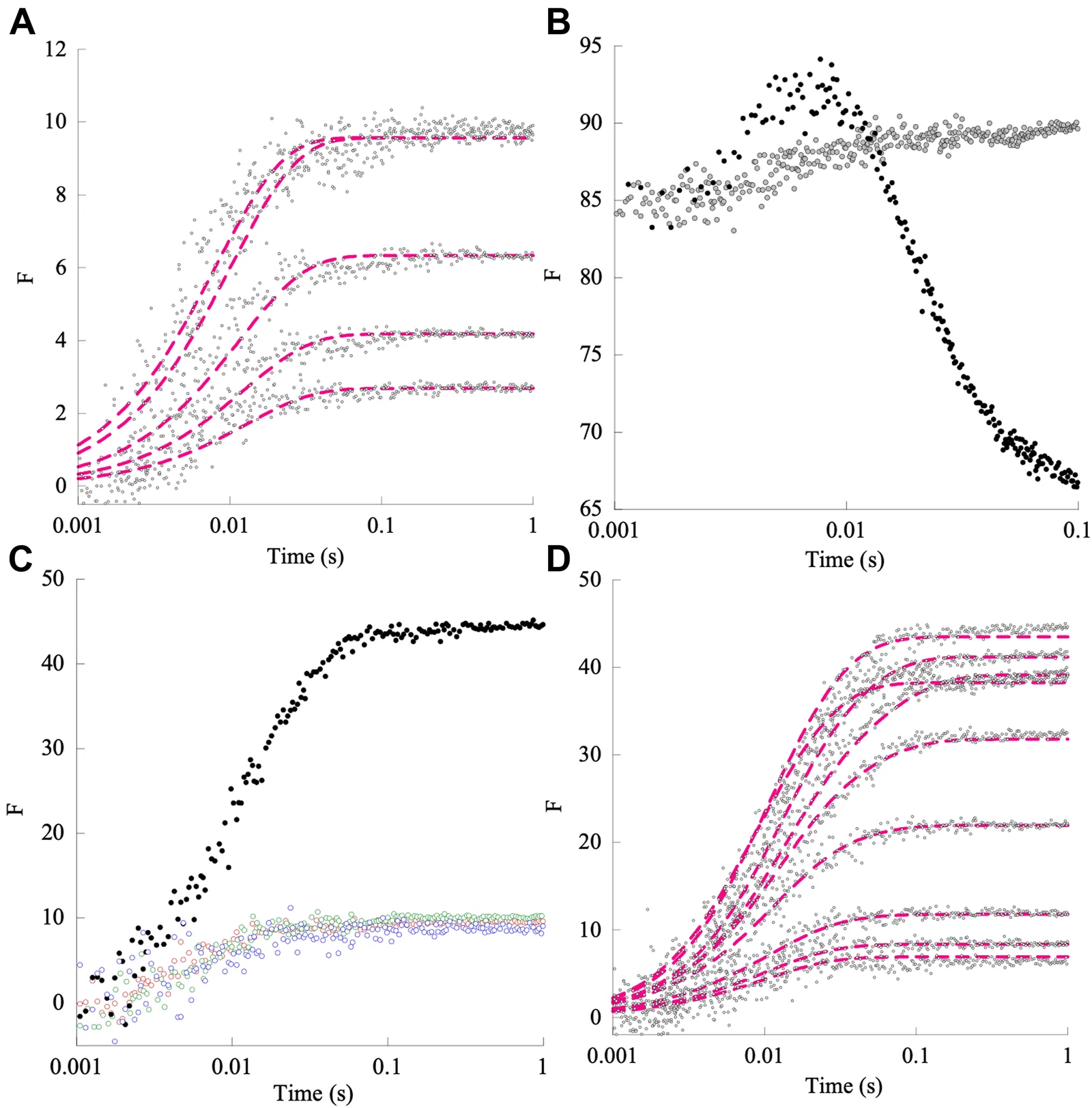
**NADPH binding assays by stopped-flow spectrometry.** Enzyme in one syringe was mixed against substrate(s) in a second syringe for each experiment. The NADPH nicotinamide ring was excited at 340 nm and fluorescence emission was measured beyond a 360 nm cut-off filter. All concentrations are the final mixed concentration in the stopped-flow cell. *A*, BfCAPADH (15 μM) binding varied NADPH from 3.75 to 60 μM. *Panel A* is one of five data sets globally fit in Kintek Explorer to the model 1. *B*, BfCAPADH (15 μM) binding NADPH (30 μM) (*gray circles*) and BfCAPADH (15 μM) oxidizing 30 μM NADPH in the presence of 1 mM agmatine and 1.25 mM aspartate semialdehyde (ASA) (*black circles*). Increasing fluorescence emission correlates to NADPH binding while loss of emission correlates to NADPH oxidation. *C*, BfCAPADH (15 μM) binding 30 μM NAPDH in the presence of no additional substrate (*red circles*), 60 μM ASA (*green circles*), 60 μM putrescine (*blue circles*), and 60 μM agmatine (*black circles*). *D*, BfCAPADH (15 μM) binding 30 μM NADPH in the presence of varied agmatine from 0.47 to 120 μM. *Panel D* is one of four data sets globally fit in Kintek Explorer to the model 2. Global fitting and FitSpace data are found in Figures S4–S8.

**Table 3:** NADPH binding rate and equilibrium constants

Constant	Rate
*k*_*1*_ (μM^−1^s^−1^)	1.0
*k*_*-1*_ (s^−1^)	61
*K*_*NADPH*_ (μM)	61
*k*_*2*_ (μM^−1^s^−1^)	5.8
*k*_*-2*_ (s^−1^)	ND
*K*_*Agm*_ (μM)	ND
*k*_*3*_ (μM^−1^s^−1^)	3.2
*k*_*-3*_ (s^−1^)	5.8
*K*_*Agm:NADPH*_ (μM)	1.8
*k*_*4*_ (μM^−1^s^−1^)	9.0
*k*_*-4*_ (s^−1^)	33
*K*_*NADP+*_ (μM)	3.6

Upon hydride transfer, the NADPH nicotinamide ring transitions from its nonaromatic, reduced state to its aromatic, oxidized form, and the corresponding excitation λ_max_ at 340 nm is lost. Thus, loss of fluorescence emission correlates with hydride transfer. When 15 μM BfCAPADH was rapidly mixed with 30 μM NADPH, 1 mM ASA, and 1.25 mM agmatine, and fluorescence emission was monitored beyond a 360 nm cut-off filter, an interesting effect was observed. The initial increase in NADPH fluorescence, correlated with binding, appeared to be much more rapid with a significant increase in slope and amplitude relative to NADPH binding in the absence of the other substrates (Fig. 5*B*). This was observed across a range of concentrations and led us to question why NADPH binding appeared enhanced.

To investigate, NADPH binding studies were conducted in the presence of ASA or putrescine or agmatine (Figs. 5*C*, S5 and S6). When 15 μM BfCAPADH was mixed with 30 μM NADPH and ASA varied from 3.75 μM to 60 μM no change was observed in the approach to binding equilibrium in comparison to NADPH binding in the absence of ASA. An equivalent result was observed when 15 μM BfCAPADH was mixed with 30 μM NADPH and putrescine varied from 0.47 μM to 120 μM. In these concentration ranges, ASA and putrescine appear to have no effect on NADPH binding rate or equilibrium. However, when 15 μM BfCAPADH was mixed with 30 μM NADPH and 60 μM agmatine a dramatic change in the approach to binding equilibrium was observed that included a large increase in fluorescence intensity, but with no shift in the emission λ_max_ (Fig. 5*C*). An increase in fluorescence quantum yield in the presence of agmatine suggests either a reduction in solvent or local side-chain quenching (possibly due to closure of a solvent channel into the active site) and/or changes to the local electrostatic environment that reduce non-radiative decay pathways, stabilizing the excited state. In essence, it suggests a conformational difference. To characterize the influence of agmatine on NADPH binding four experiments were conducted wherein 15 μM BfCAPADH was mixed with NADPH varied from 0.47 to 30 μM and with agmatine varied between 0.47 μM and 2 mM (Figs. 5*D* and S7). These data were globally fit to model 2 in which agmatine associates with BfCAPADH first and NADPH binds second. The resulting FitSpace (12) was well constrained overall but did not define the lower limit of *k*_*-2*_, the off rate for the BfCAPADH:agmatine complex. The calculated parameter for agmatine association was *k*_*2*_ = 5.8 μM^−1^s^−1^. Agmatine-dependent binding of NADPH demonstrated a 3.2-fold increase in the on rate to *k*_*3*_ = 3.2 μM^−1^s^−1^ and a 10.5-fold decrease in the off rate to *k*_*-3*_ = 5.8 s^−1^. Accordingly, the equilibrium value decreased 34-fold from *K*_*NADPH*_ = 61 μM to *K*_*Agm:NADPH*_ = 1.8 μM (Table 3). This suggests that agmatine is kinetically preferred to bind first, followed by NADPH, in contrast with the nearest characterized homolog, saccharopine dehydrogenase, which binds NADH first and the amine nucleophile last.

We considered the possibility that putrescine might induce NADPH binding at a higher putrescine concentration. When 15 μM BfCAPADH was mixed with 2 mM putrescine and NADPH varied from 0.47 μM – 30 μM we observed a smaller enhancement in NADPH binding and minimal increase in emission intensity. We fit these data to model 1 (one-step equilibrium) and model 2 (two-step equilibrium). Model 2 did not result in an improved fit, and the rate constants were poorly constrained. The fit to model 1 suggests that putrescine, at 2 mM, is able to enhance the NADPH on rate to 2.7 μM^−1^s^−1^, but the off rate only decreases to 29 s^−1^ indicating 6-fold weaker NADPH binding and at a much higher concentration (2 mM) (Fig. S6, *B*–*D*).

Having observed a stabilization of CAPADH in the presence of NADP^+^, we tested the ability of NADP^+^ to compete with NADPH for occupancy of the active site. In these experiments, 15 μM BfCAPADH was mixed with NADPH at 30 μM or 120 μM and NADP^+^ varied from 3.75 μM to 60 μM. As NADP^+^ was increased, the fluorescence amplitude decreased indicating that NADP^+^ was occupying the binding site and more NADPH remained unbound and in solution. These data were fit to a competitive one-step equilibrium binding model (model 3) in Kintek Explorer (11) (Fig. S8). The resulting FitSpace (12) was well constrained. The calculated parameters for NADP^+^ binding were *K*_*NADP*_^*+*^ = 3.6 μM, *k*_*4*_ = 9.0 μM^−1^s^−1^ and *k*_*-4*_ = 33 s^−1^ (Table 3).

### Overview of CAPADH apo X-ray structures

Apo X-ray crystal structures of ClCAPADH and BfCAPADH, which share 64% sequence identity, were solved at 2.50 Å and 2.91 Å respectively and deposited in the Protein Data Bank (PDB: 9YQ9 and 36GY). Data were collected on beamline 12-1 and 12-2 at the Stanford Synchrotron Radiation Lightsource (SSRL) and data collection and refinement statistics and model composition analysis are found in Tables 4 and S3. Both ClCAPADH-Apo and BfCAPADH-Apo were solved by molecular replacement using BfCAPADH-NADP^+^ (PDB: 8DEB) chain A as a model. It was immediately evident, following the first round of refinement for both structures, that significant conformational differences existed for the position of the dimerization domain and for three active site loops (β 5-α 5, β 7-α 8 and α 11-β 15) (Fig. 6). CAPADH adopts an “M” shape in which the dimer is formed at the point of the M by a 987 Å (Cl) or 1040 Å^2^ (Bf) interface as determined by PDBePISA (17). The overall domain structure of CAPADH has been described previously for *B. fragilis* (7) and *Helicobacter pylori* (18) and is composed of NADPH-binding (Fig. 6 – green), catalytic (Fig. 6 – blue), and dimerization domains (Fig. 6 – yellow). The angle between the dimeric interface and the “legs” of the M (the NADPH-binding and catalytic domains) was 8° and 5° greater for Cl- and BfCAPADH-Apo respectively when compared to BfCAPADH-NADP^+^. The observed conformational differences led us to consider whether the crystalline structures were representative of the conformation for these proteins in solution.

**Figure 6:**
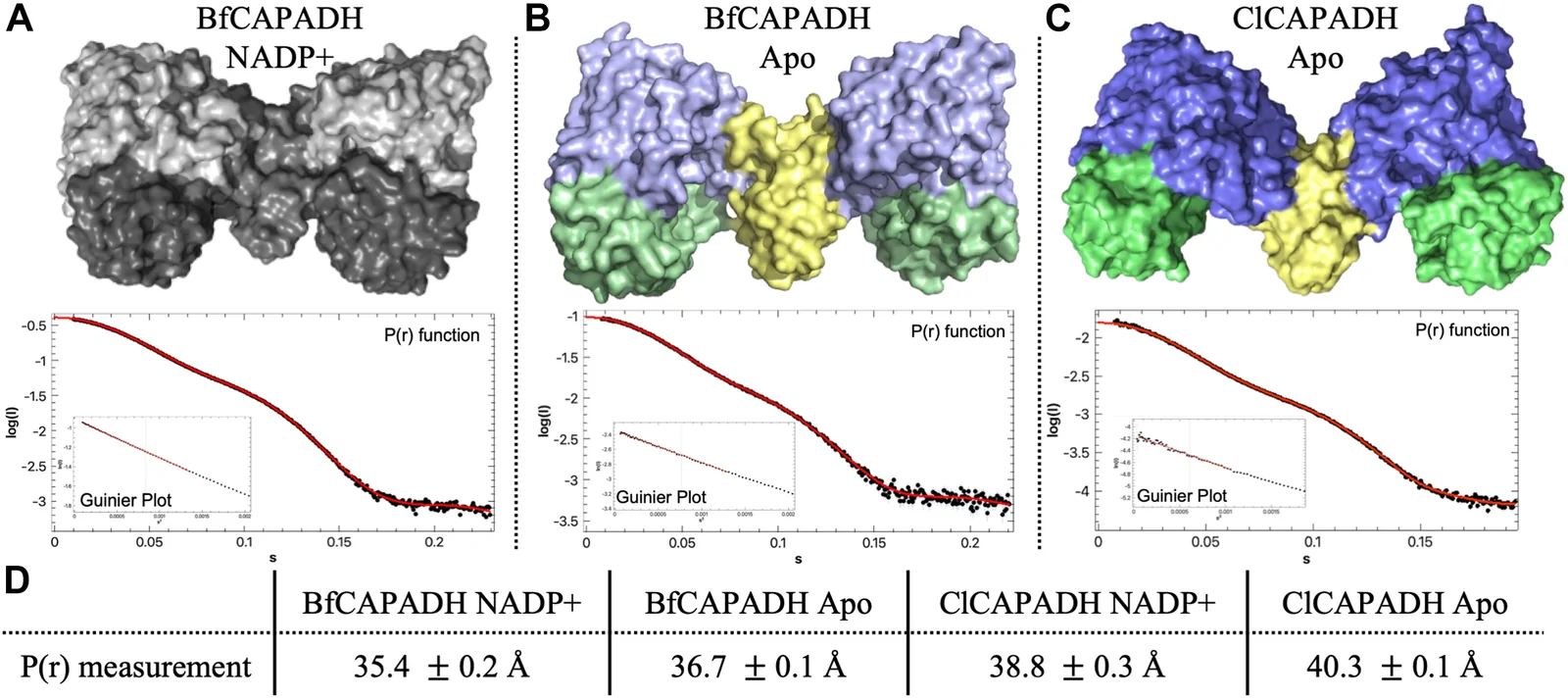
**CAPADH apo structural overview and SAXS comparison.***A*, BfCAPADH – NADP^+^ (PDB:8DEB) is compact with a radius of gyration (*R*_*g*_) of 35.4 Å as determined by small angle x-ray scattering (SAXS). *B*, BfCAPADH – Apo (PDB: 36YL) adopts a more relaxed conformation in crystalline form and in solution with a *R*_*g*_ = 36.7 Å. *C*, ClCAPADH – Apo (PDB: 9YQ9) is most extended with a *R*_*g*_ = 40.3 Å. The corresponding SAXS P(r) function and Guinier plots are shown below each structure and are deposited in SASBDB as described in methods. Domain coloring: *green* – NADPH binding domain; *blue* – catalytic domain; *yellow* – dimerization domain. *D*, radius of gyration (*R*_*g*_) values. P(r) measurements were experimentally determined from the P(r) function plots of solution scattering data. CAPADH, carboxyaminopropylagmatine dehydrogenase; ClCAPADH, Clostridium leptum carboxyaminopropylagmatine dehydrogenase; SASBDB, Small Angle Scattering Biological Data Bank; SAXS, small angle X-ray scattering.

### SAXS of CAPADH

SAXS experiments were conducted on beamline 4-2 at the SSRL to examine the solution properties of Bf- and ClCAPADH in their apo states and in the presence of NADPH and NADP^+^. Protein was purified by nickel affinity and size exclusion chromatography, as described in methods, and dilution series were prepared in matched buffers ranging in concentration from 9.0 to 0.3 mg/ml for BfCAPADH and 4.5 to 0.3 mg/ml for ClCAPADH. NADPH and NADP^+^ were added at concentrations 10-fold above their respective *K*_*m*_ values. Protein samples were observed to be monodisperse leading to high quality data sets. Data were deposited in the Small Angle Scattering Biological Data Bank.

Radius of gyration ((21) *R*_*g*_) was calculated from solution scattering data from Guinier and P(r) function plots (Figs. 6 and S9). The scattering data reflected the trend found in the crystal structures where BfCAPADH-NADP^+^ forms a compact crystal structure with a SAXS *R*_*g*_ = 35.4 ± 0.2 Å, while BfCAPADH-Apo is more extended with a SAXS *R*_*g*_ = 36.7 ± 0.1 Å. Likewise, ClCAPADH-Apo is the most extended crystal structure (SAXS *R*_*g*_ = 40.3 ± 0.1 Å), while the ClCAPADH-NADP^+^ SAXS data indicate that it is more compact in solution (*R*_*g*_ = 38.8 ± 0.3 Å) (Fig. 6). Taken together these data confirm that CAPADH adopts differing conformational states, that the presence of NADP^+^ or NADPH causes both proteins to adopt a more compact domain arrangement, and that the crystal structures are representative of the conformational trends observed in solution.

### Loop dynamics

Three loops (β5-α5, β7-α8 and α11-β15) overlay the active site and undergo significant conformational rearrangement between apo and NADP^+^-bound states (Figs. 7, *A*–*C*, S10 and S11). In the BfCAPADH-NADP^+^ structure, the β7-α8 loop is positioned between the α11-β15 loop and the dimerization domain. This allows N181 of the β7-α8 loop to form hydrogen bonds that stabilize NADP^+^ against the Rossmann-like fold of the NADPH-binding domain, which correlates with the increased stability observed in thermal shift assays with NADP^+^. In the BfCAPADH-Apo structure, residues 174 to 178 of the β7-α8 loop are positioned within the NADPH binding channel, forming a hydrogen bond with G11, and must be displaced to allow the association of NADPH. Thus, while BfCAPADH-Apo has a more extended domain structure, the NADPH binding site is occluded. Repositioning the β7-α8 loop to allow NADPH binding requires displacement of the α11-β15 loop. Based on BfCAPADH-NADP^+^, this displacement is accompanied by a secondary structural rearrangement wherein residues 295 to 299 add to helix α11 which shifts 13.5 Å to accommodate the motion of β7-α8. Interestingly, the ClCAPADH-Apo structure is even more extended, but it does not exhibit the secondary structural rearrangements found in BfCAPADH-Apo. Rather, the global secondary structure seen in BfCAPADH-NADP^+^ is largely retained in ClCAPADH-Apo. The NADPH binding site is still occluded, but by more subtle repositioning of L84, P85, and N107 that would cause steric clash with NADPH (Fig. 7*D*). It is possible that Cl- and BfCAPADH-Apo structures represent two different states that are sampled by both proteins. Alternatively, it is possible that the apo structures represent structural distinctions between these species. The ClCAPADH-Apo structure suggests a means by which NADPH could bind in the absence of agmatine, albeit less efficiently, as only a minor rearrangement of residues 83 to 88 would be necessary to accommodate NADPH.

**Figure 7:**
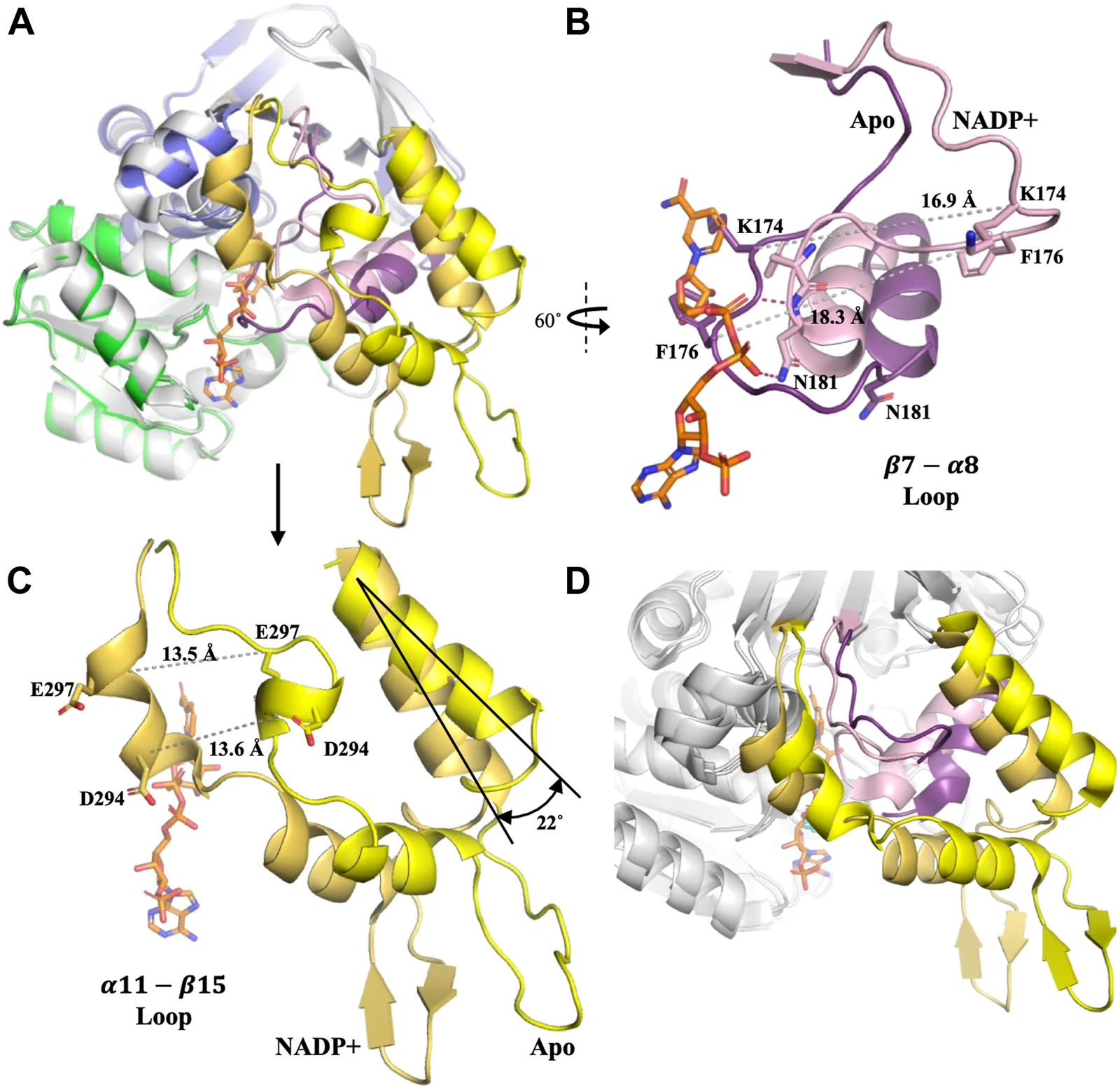
**Loop dynamics.***A*, overlay of BfCAPADH structures. BfCAPADH with NADP^+^ bound. *Orange* – NADP^+^. *Gray* – NADPH and catalytic domains. *Pink* - β7- α8. *Gold* - α11-β15 loop and dimerization domain. BfCAPADH-apo. *Green* – NADPH binding domain. *Blue* – catalytic domain. *Violet* - β7-α8. *Yellow* - α11-β15 loop and dimerization domain. *B*, comparison of β7- α8 loop positions. BfCAPADH-NADP^+^ - *pink*. BfCAPADH-apo – *purple*. The NADP^+^ binding site is occluded by K174 and F176 in the apo state. *C*, comparison of α11-β15 loop positions. BfCAPADH-NADP^+^ - *gold*. BfCAPADH-apo – *yellow*. A 22° hinge movement is observed. *D*, overlay of Bf-NADP^+^ and ClCAPADH apo structures. ClCAPADH-apo – *gray*/*yellow*/*violet*. BfCAPADH-NADP^+^ - *gray*/*gold*/*pink*. The α11-β15 (*yellow*/*gold*) and β7- α8 (*pink*/*violet*) loops in ClCAPADH-apo have similar secondary structure to BfCAPADH-NADP^+^, but a hinge movement increases the angle between the NADPH-binding and catalytic domains and the dimerization domain. ClCAPADH, Clostridium leptum carboxyaminopropylagmatine dehydrogenase.

### Agmatine binding site predictions

We reasoned that agmatine might induce the conformational change in the β7-α8 loop that clears it from the NADPH binding site. To evaluate the structural role of agmatine in the CAPADH catalytic cycle, it was essential to consider where agmatine might bind. Efforts are ongoing to solve a CAPADH structure with agmatine bound. Herein, we used structural sequence alignments to form a hypothesis about the residues that allow selectivity for agmatine, putrescine or diaminopropane and Boltz-2 protein-ligand modeling to computationally evaluate this hypothesis. Five homologs were selected for sequence alignment analysis. They were Bf- and ClCAPADH (agmatine), CASDH homologs (putrescine) from *Brucella abortus* and *A. tumefaciens* and a CANSDH homolog (diaminopropane) from *Vibrio cholera*. Structural alignments were performed using Expresso on the T-COFFEE server and were annotated in ESPript 3.2 (Fig. S11).

The BfCAPADH-NADP^+^ structure has continuous electron density for the β5-α5 (residues 101–120 in BfCAPADH) loop in a conformation that excludes access to the active site; however, the β5-α5 loop adopts multiple conformations across the Cl- and BfCAPADH-Apo structures and often exhibits weak or no density. This indicates that the β5-α5 loop “opens” to provide a possible path for substrate entry or product egress and it encodes a negatively charged motif that could act to stabilize the positively charged guanidinium or primary amine group found in agmatine, putrescine and diaminopropane. Sequence and structural alignments demonstrated distinctions within this motif between the three homolog groups (Fig. 8). E107 is conserved in CAPADH and CASDH homologs but is a valine in CANSDH. Spatially adjacent residues N105 and E229 in CAPADH are substituted with isoleucine and aspartate in the CASDH and valine and aspartate in CANSDH. We reasoned this motif formed the substrate binding site and that E107 could form an ionic interaction with N^ω^ on either agmatine or putrescine and that N105 and E229 could complete the interactions with N^4^ and N^ω’^ of the guanidinium group while an isoleucine would the fill space not occupied by the shorter putrescine substrate. When a Boltz-2 model was generated with the CAPA product molecule, the guanidinium group was coordinated by E229, N105 and E107 as predicted. C3 of the 3-amino-3-carboxypropyl moiety of CAPA was positioned for hydride transfer at 3.5 Å from C6 of the nicotinamide ring. The adjacent N^4^ was 3.1 Å from H228, the residue predicted to act as the active site base. Additional models using CSP and CNSP placed C3 in the same position, while the C7 (CSP) and C6 (CNSP) carbons are in a shallower position allowing D229 (CASDH) and S106 (CANSDH) to coordinate the terminal amine. If this hypothesis is correct, the β5-α5 loop is actually a substrate recognition loop allowing this family of dehydrogenases to select between agmatine, putrescine, and diaminopropane as initiating substrates.

**Figure 8:**
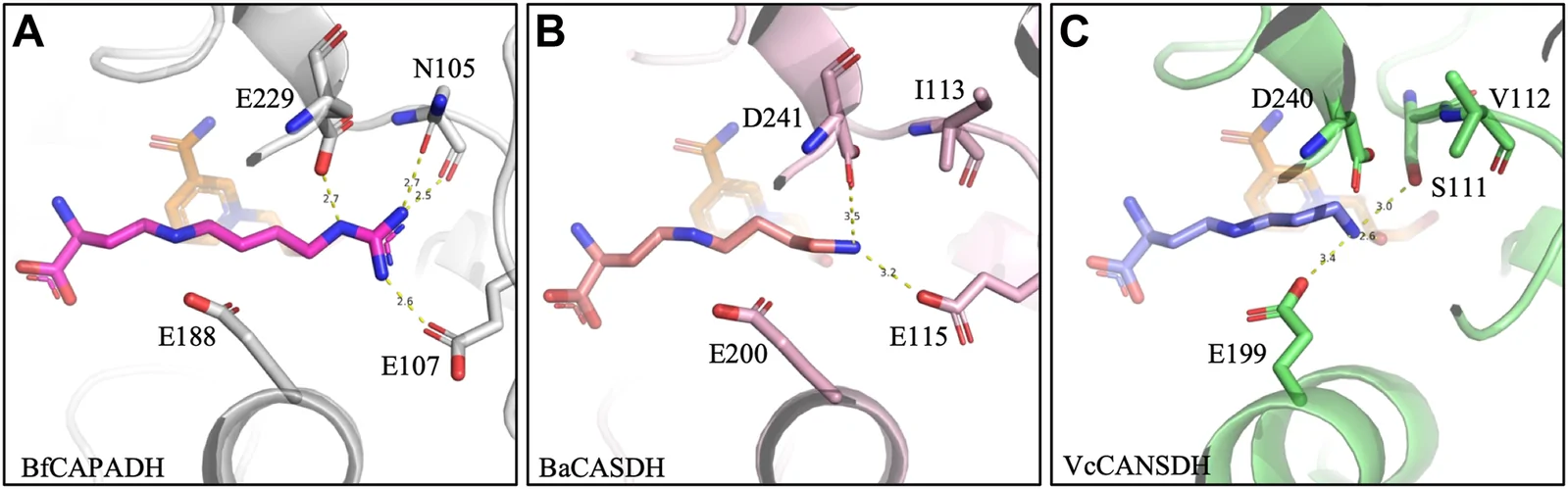
**Boltz-2 substrate binding predictions.** NAPDH – *orange*. *A*, BfCAPADH. Carboxyaminopropylagmatine – *magenta carbons*. *B*, BaCASDH (*Brucella abortus*). Carboxyspermidine – salmon. *C*, VcCANSDH (*Vibrio cholera*). Carboxynorspermidine – *blue*.

### Hypothetical mechanism for agmatine-induced NADPH binding

The predicted binding site for agmatine was examined by comparing the Boltz-2 model with the BfCAPADH-Apo structure to develop a hypothetical mechanism by which agmatine binding could induce the conformational switch necessary to enhance NADPH binding (Fig. 9). In the apo structure, the β5-α5 loop adopts multiple conformations and often exhibits weak electron density, α8 is too far to interact with CAPA or the β5-α5 loop, and K174 of the β7-α8 loop occupies the site where the nicotinamide ring would bind. When agmatine binds, it would stabilize the β5-α5 loop through hydrogen bonding with the guanidium group, and N^1^ of agmatine would repel K174 of the β7-α8 loop. Repelling K174 could cause the β7-α8 loop to flip out of the NADPH binding site, moving α8 closer and placing N181 in position to hydrogen bond with the nucleotide phosphates of an incoming NADPH. As α8 is drawn closer, E188 would form an electrostatic interaction with N^1^ and the β5-α5 loop would be further stabilized by a bridge between E107 and R187 establishing a ternary complex between enzyme, NADPH and agmatine. Thermal stability data and the decreased off rate for NADPH in the presence of agmatine suggest an energy state that is lower than both the Apo and NADP^+^ bound conformations. Thus, the positions of the β7-α8 and α11-β15 loops observed in the BfCAPADH-NADP^+^ structure may differ from those for the ternary complex or the Boltz-2 model. Additional structural data and/or molecular dynamic simulations are necessary to evaluate the conformational state of the ternary complex and mutagenesis studies are needed to confirm our hypothesis of substrate recognition.

**Figure 9:**
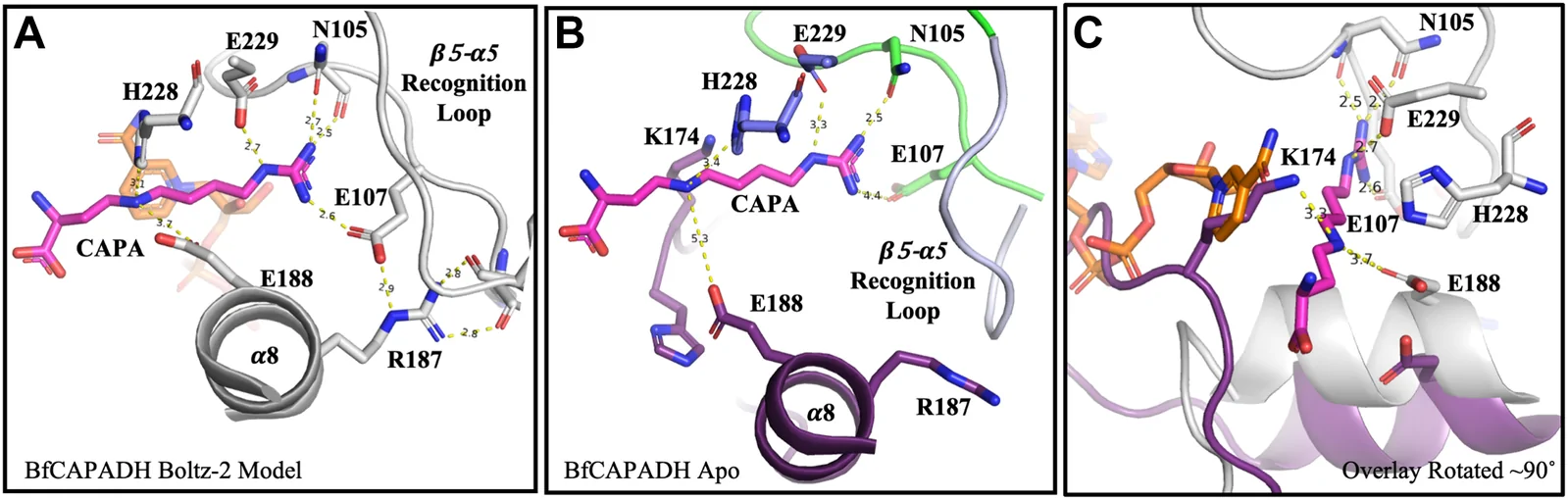
**Structural hypothesis for agmatine induced NADPH binding.***A*, BfCAPADH Boltz-2 model generated with NADPH (*orange*) and carboxyaminopropylagmatine (CAPA) (*magenta*). E107 and R187 stabilize the recognition loop in a “closed” position. *B*, BfCAPADH-apo crystal structure with CAPA from the Boltz-2 model superimposed within the active site. The *green polyamine recognition loop* has incomplete electron density in chain C (*green*) and continuous electron density in chain A (*gray*). R187 has no side chain density visible past C β and was modeled in a favorable rotameric position. *C*, overlay of BfCAPADH Boltz-2 model and BfCAPADH-Apo. Rotated ∼90°. Color pattern is retained from panels A and B. K174 of the β7-α8 loop clashes with NADPH in the apo structure, but has cleared the NADPH binding site in the Boltz-2 model.

## Conclusion

This work establishes that *B. fragilis* initiates ASA-dependent spermidine biosynthesis with agmatine rather than putrescine and corroborates the same finding by Li *et al.* for *C. leptum*, demonstrating that these organisms encode CAPADHs and decarboxylases rather than CSP enzymes. Together with recent reports from other bacterial species, these results indicate that ASA dependent spermidine biosynthesis, using agmatine as the initiating substrate, is more broadly distributed than previously appreciated and suggests that annotation of members of this enzyme family should be reconsidered. Beyond defining substrate specificity, our kinetic and structural studies provide a mechanistic framework for understanding the role of agmatine in CAPADH catalysis. The data support a model in which agmatine binding promotes a conformational transition that accelerates NADPH association and stabilizes the ternary complex, thereby coupling substrate recognition to productive cofactor binding. Structural analysis identifies a putative polyamine recognition loop that may distinguish between agmatine, putrescine, and diaminopropane as substrates. These findings expand our understanding of the diversity of microbial polyamine biosynthesis and provide a foundation for identifying agmatine-dependent pathways from sequence alone. Future structural characterization of ternary enzyme-substrate complexes and targeted mutagenesis of the proposed recognition residues should further define the conformational changes that underlie substrate selectivity and catalysis.

## Experimental procedures

### Reagents

Putrescine (1,4 diaminobutane), PLP monohydrate, and ^13^C spermidine (spermidine-(butyl-^13^C4) trihydrochloride) were obtained from Sigma-Aldrich, agmatine was obtained from Thermo Fisher Scientific, nicotinamide adenine dinucleotide phosphate (NADP^+^) was obtained from CoALA Biosciences, NADPH was obtained from EMD Millipore, and ASA was synthesized as previously described (7, 19, 20, 21).

### Expression and purification of BfCAPADH, ClCAPADH, and BfCAPADC

The expression and purification of BfCAPADH (WP_005801052.1) and ClCAPADH (EDO61991.1) followed our previously published methods. The gene sequence for BfCAPADC, NCBI accession number CAH08254.1, was codon optimized for expression in *Escherichia coli* by GenScript and ligated into a pET-29b vector containing and C-terminal hexahistidine affinity tag. The resulting plasmids were transformed into New England Biolabs BL21 (D3) cells for overexpression.

BfCAPADC was expressed in baffled flasks with 1 L of LB Miller broth containing 50 μg/ml kanamycin, inoculated with 10 ml of overnight culture, and grown to A_600_ of 0.6 to 0.8 at 37 °C (∼3 h). The cultures were induced with 200 μl 1 M IPTG and grown for 19 h at 37 °C. Cells were centrifuged at 3025 rcf for 10 min. The pellet was resuspended in 10 ml of buffer A: 25 mM Tris pH 8, 300 mM NaCl, and 50 mM imidazole. Cells were lysed with a Branson SFX 150 sonicator at 75% amplitude using a 15 s pulse, 45 s pause cycle. Lysates were centrifuged at 23,700 rcf for 45 min and the supernatants injected onto a nickel-chelating Sepharose column (Cytiva 6 Fast-Flow resin) equilibrated with five column volumes of buffer A. Protein eluted in 100% buffer B (25 mM Tris pH 8, 300 mM NaCl, and 300 mM imidazole) and flowed directly onto a Cytiva HiPrep 26/10 desalting column preequilibrated with buffer C: 25 mM Tris pH 8, 300 mM NaCl.

### PLP saturation of BfCAPADC

Following elution from the desalting column BfCAPADC was scanned from 260 to 500 nm on an Agilent Cary 60 UV-Vis spectrophotometer to observe the PLP absorbance baseline (peaks were evident at 335 and 420 nm). PLP was mixed with the protein sample to a concentration of 200 μM and incubated on ice for 20 min. The sample was injected onto a Cytiva HiPrep 26/10 desalting column preequilibrated with buffer C to separate the unbound excess PLP. The resulting protein sample typically demonstrated a 30% increase in absorbance at 420 nm indicative of PLP loading. The concentration of BfCAPADC was calculated from the peak at 280 nm (ϵ = 56,840 M^−1^ cm^−1^) and used for assay calculations. Protein was concentrated to 5 to 10 mg/ml using an Amicon 10 kDa concentrator and was stored at −80 °C after flash freezing aliquots in liquid nitrogen. A typical preparation yields 30 mg of BfCAPADC per liter of culture.

### CSP and CAPA preparation

CSP and CAPA were prepared enzymatically using BfCAPADH. The reaction was monitored to completion by observing NADPH oxidation on an Agilent Cary 60 UV-Vis spectrophotometer at 340 nm. The reaction buffer was 25 mM Tris, pH 7.5 and reaction temperature was 25 °C. To prepare CAPA, the reaction mix contained 1 μM BfCAPADH, 100 to 150 μM NADPH, 1 mM ASA, and 1.25 mM agmatine. To prepare CSP, the reaction mix contained 4 μM BfCAPADH, 200 μM NADPH, 2 mM ASA, and 20 mM putrescine. The amount of CSP or CAPA produced was calculated to be equal to the NADPH consumed (NADPH ϵ = 6220 M^−1^ cm^−1^). Varied concentrations of CSP or CAPA were made by serial dilution in 25 mM Tris pH 7.5.

### Coupled mass spectrometry assay of BfCAPADH/BfCAPADC activity

CSP (50 μM) was added to a 100 μl reaction mixture containing 1 μM BfCAPADC in 25 mM Tris pH 7.5. The reaction was incubated for 1 h at 25 °C, quenched with 100 μl trichloroacetic acid (4%), vortexed for 5 min, and centrifuged at 10,000 rcf for 1 min. A total of 150 μl of the supernatant was combined with 800 μl ultrapure water, 125 μl 1 M sodium carbonate pH 9, and 25 μl isobutyl chloroformate for derivatization (Fig. 10). The mixture was incubated for 15 min at 35 °C and centrifuged at 10,000 rcf for 1 min. A total of 900 μl of supernatant was combined with 900 μl of diethyl ether, inverted, and the top layer was extracted by micropipetting. The diethyl ether was evaporated (10–15 min) in a Thermo Scientific Savant SpeedVac. Dried samples were resuspended in 100 μl methanol, vortexed for 15 s and analyzed by LC-MS/MS.

**Figure 10:**
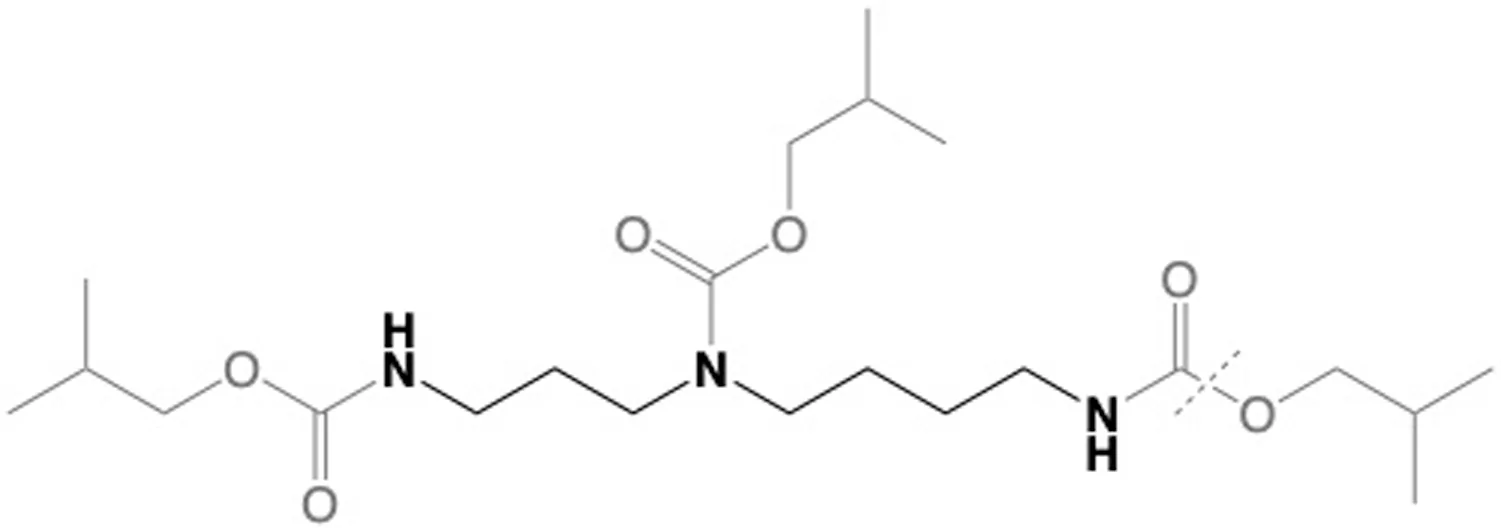
Isobutyl chloroformate derivatized spermidine.

Samples were analyzed on an Agilent 6460 Triple Quad LC/MS with an Agilent Eclipse Plus C18 column (4.6 × 150 mm, 5 μm particle size) at 35 °C. The flow rate was 0.5 ml/min and the injection volume was 5 μl. Mobile phase A consisted of 0.1% formic acid in 5% ACN and mobile phase B consisted of 0.1% formic acid in 95% ACN. A gradient elution used the following sequence: 0 to 6 min at 50% B, 6 to 10 min from 50% to 95% B, 10 to 15 min at 50% B. An Agilent Jet Stream ion source was used in positive ion mode with the following parameters: 3500 V capillary voltage, 300 °C source temperature, 5.0 l/min N_2_ drying gas flow rate, and 45 psi nebulizer pressure. MS1 scanned for the following [M + H]^+^ precursor ions: spermidine – 446 *m/z*, putrescine - 289 *m/z*, ^13^C spermidine – 450 *m/z*. Samples were fragmented at 135 eV with collision-induced dissociation using ultra high purity nitrogen gas. The data were acquired using Agilent MassHunter Workstation and EIC peak areas were calculated based on the following [M + H]+ product ion values: spermidine - 372 *m/z*, putrescine - 215 *m/z*, and ^13^C spermidine - 376 *m/z*.

### Steady state kinetics of the forward reaction

Initial rates were measured by observing NADPH oxidation as a decrease in absorbance at 340 nm using a TgK scientific stopped-flow spectrometer at 24 °C. The reaction buffer was 25 mM Tris pH 7.5. For each reaction, 50 μl from a syringe containing enzyme and substrates held in excess was rapidly mixed 1:1 with 50 μl from a second syringe containing varied concentrations of NADPH, ASA, or agmatine. The master mix for varied ASA contained 1 μM BfCAPADH or 0.2 μM ClCAPADH, 150 μM NADPH, and 1.25 mM agmatine. ASA was prepared separately in concentrations from 10 mM to 0.02 μM to initiate the reaction. The master mix for varied agmatine contained 1 μM BfCAPADH or 0.2 μM ClCAPADH and 150 μM NADPH. Agmatine was prepared separately in concentrations from 5 mM to 0.6 μM and 1 mM ASA was added to each agmatine dilution immediately before mixing to initiate the reaction as we have observed degradation of enzyme activity when left in the presence of ASA. The master mix for varied NADPH contained 1 μM BfCAPADH or 0.2 μM ClCAPADH and 1.25 mM agmatine. NADPH was prepared in concentrations from 200 μM to 0.1 μM and 1 mM ASA was added as described previously. To determine kinetic parameters, secondary plots of initial rates were fit in Kaleidagraph to the Michaelis-Menten equation expressed in terms of *k*_*cat*_ (Equation 1).

(1) $$\frac{V_{o}}{\left[E\right]}=\frac{k_{cat\;}\left[S\right]}{K_{m}+\left[S\right]}$$

Each experiment was performed in triplicate and error was calculated as the SD of parameters determined from each separate trial.

### Multiwavelength stopped-flow spectrometry coupled enzyme assay

A transient state stopped-flow spectrometry assay was designed to test the ability of BfCAPADC to convert CSP and CAPA to spermidine or APA, respectively. A TgK Scientific stopped-flow spectrometer with a KinetaScan CCD detector was used for rapid absorbance scanning from 305 to 550 nm using a 3.2 s data collection time in log mode with a 1.6 ms scan integration time. The reaction buffer was 25 mM Tris pH 7.5 and the reaction temperature was 4 °C. The final concentrations in the stopped-flow cell after mixing are reported. CSP and CAPA were prepared enzymatically as described previously. CSP or CAPA were serially diluted to 50 μM, 25 μM, 12.5 μM, and 6.25 μM in reaction buffer and rapidly mixed with 25 μM BfCAPADH. Conversion of substrate to product was observed as changes in the PLP absorbance spectra. Data for CAPA were analyzed by single value decomposition in Kintek Explorer. Three components were selected and modeled using a two-step, irreversible model. Each experiment was conducted twice. Data for CSP demonstrated minimal and inconsistent spectral changes over timescales up to 314 s providing no clear pattern for single value decomposition analysis.

### Thermal shift assay

Thermal shift assays were conducted on a Bio-Rad 384CFX real-time thermal cycler. Fluorescence intensity of a Sypro Orange (Thermo Fisher Scientific) probe was measured while ramping temperature from 10 °C to 95 °C in 1 °C increments. A total of 40 μM CAPADH was prepared in 25 mM Tris pH 8 (ClCAPADH) or 25 mM KPi buffer pH 8 (BfCAPADH) and Sypro Orange was added to a concentration of 5X. Ligand concentrations were calculated such that the final reaction concentration was in 10-fold excess over the measured *K*_*m*_ values for that ligand as reported in this article and previously (7). Ligand stock solutions were prepared in ultrapure water such that 2 μl of ligand would yield the required final concentrations. The final reaction volume was 20 μl consisting of 5 μl of CAPADH/Sypro solution, 5 μl of reaction buffer, 2 or 4 μl of ligand, and 6 or 8 μl of ultrapure water. Reactions were performed in triplicate with six technical replicates in each experiment. *T*_m_ values were determined by visual inspection of the peak of first derivative plots of the melting curves.

### Stopped-flow binding assays

The binding of NADPH by BfCAPADH was measured as an increase in fluorescence emission beyond a 360 nm cut-off filter using an excitation wavelength of 340 nm on a TgK stopped-flow spectrometer at 24 °C. BfCAPADH was mixed with NADPH alone, or with additional substrates. All concentrations given are the mixed concentration in the stopped-flow cell. Fluorescence emission was measured for 1 s in log timescale mode. For each experiment, three separate technical replicates were collected at each concentration and averaged.

### NADPH binding

A total of 15 μM BfCAPADH (experiment 1 and 3–5) or 25 μM (experiment 2) was mixed with NADPH over a series of five experiments at the following concentrations: experiment 1 to 3.125 to 50 μM; experiment 2 to 10 to 40 μM; experiment 3 to 3.75 to 60 μM; experiments 4 and 5 – 0.56 to 36 μM. The data were globally fit to model 1in KinTek Explorer (E = BfCAPADH, N=NADPH, and EN = binary complex).

$$\;\text{Model}\;1.\;E\;\begin{array}{c} k_{1}\\ ⇋\\ k_{-1} \end{array}\text{EN}$$

### NADPH binding in the presence of agmatine

A total of 15 μM BfCAPADH was mixed with NADPH and agmatine in the following concentrations. Experiment 1: 0.47 to 7.5 μM NADPH and 2 mM agmatine; experiment 2: 30 μM NADPH and 3.75 to 60 μM and 1.25 mM agmatine; experiment 3: 30 μM NADPH and 0.47 to 120 μM agmatine; experiment 4: 0.47 to 30 μM NADPH and 2 mM agmatine. The data were globally fit to model 2 in KinTek Explorer (E = BfCAPADH, N=NADPH, A = agmatine, EA = binary complex, and EAN = ternary complex).

$$\;\text{Model}\;2.\;\text{EN}\;\begin{array}{c} k_{1}\\ ⇋\\ k_{-1} \end{array}E\;\begin{array}{c} k_{2}\\ ⇋\\ k_{-2} \end{array}\text{EA}\;\begin{array}{c} k_{3}\\ ⇋\\ k_{-3} \end{array}\text{EAN}$$

### NADP^+^ inhibition of NADPH binding

The competition of NADP^+^ for the NADPH binding site was observed by mixing 15 μM BfCAPADH with 30 μM or 120 μM NADPH and varied concentrations of NADP^+^ from 3.75 to 60 μM in two separate experiments. The data were globally fit to model 3 in KinTek Explorer (E = BfCAPADH, N=NADPH, P=NADP^+^, and EN and EP = binary complexes).

$$\;\text{Model}\;3.\;\text{EN}\;\begin{array}{c} k_{1}\\ ⇋\\ k_{-1} \end{array}E\;\begin{array}{c} k_{4}\\ ⇋\\ k_{-4} \end{array}\text{EP}$$

### NADPH binding in the presence of putrescine

BfCAPADH at 15 μM was mixed with 0.47 to 30 μM NADPH and 2 mM putrescine. This experiment was repeated twice. The data were globally fit to model 1 in KinTek Explorer as the fit was not altered by including the concentration of putrescine in the model. BfCAPADH at 15 μM was mixed with 30 μM NADPH and 0.47 to 120 μM putrescine. These data showed no concentration dependency and were equivalent to binding of NADPH in the absence of putrescine.

### NADPH binding in the presence of ASA

A total of 15 μM BfCAPADH was mixed with 30 μM NADPH and 3.75 to 60 μM ASA. These data showed no concentration dependency and were equivalent to binding of NADPH in the absence of ASA.

### CAPADH crystallization

All crystals were grown in sitting drop trays with a 1 μl: 1 μl ratio of protein and well solution at 4 °C. BfCAPADH crystals were grown using 4 mg/ml protein in a well solution of 0.15 M ammonium citrate dibasic and 22% PEG 3350. Crystals grew within 3 days in a diamond morphology and were cryoprotected in well solution supplemented with 25% (v/v) glycerol. ClCAPADH crystals were grown using 5 mg/ml protein in a well solution of 0.85 M lithium chloride, 0.1 M Tris HCl pH 7.5, and 28% PEG 8000. Crystals grew in 2 to 5 days in a flower morphology of thin sheets and were cryoprotected in well solution supplemented with 25% (v/v) glycerol. The crystals were looped and flash-frozen in liquid nitrogen prior to data collection.

### ClCAPADH data collection and structure determination

Diffraction data for ClCAPADH were collected remotely using Blu-ICE (22) on beamline 12-2 at the SSRL. A total of 360° of data were collected at a wavelength of 0.9795 Å with 0.15° oscillations and 0.2 s exposures at a temperature of 100 K. The detector distance was 385 mm. Statistics for data collection and refinement data are listed in Table 4. The data were processed to 2.50 Å in XDS (23). The ClCAPADH structure was solved by molecular replacement using Phenix Phaser (24) with 8DEB, Chain A as a model. The resulting log-likelihood gain was 885.3 with a TFZ score of 20.3. Initial model building was done in Phenix Autobuild, which placed 1414 of 1596 residues with R_free_ = 33.98 and R_work_ = 27.61. Rounds of model building and refinement were completed in Coot and Phenix Refine (25). Waters were placed by Phenix Refine and corrected manually. The finished model was refined to R_free_ = 24.46 and R_work_ = 18.87. Ramachandran analysis was performed by MolProbity, showing 97.35% favored conformations with 0.07% outliers.

**Table 4:** Data collection and refinement statistics

Parameter	ClCAPADH-apo	BfCAPADH-apo
Data collection		
Space group	P1	P6_5_
Unit cell (Å)	*a* = 43.9, *b* = 65.2, *c* = 156.5	a = 117.7, b = 117.7,c = 297.6, α = 90, β = 90, γ = 120
Resolution range (Å)	38.86–2.5	39.24–2.91
Completeness (%)	92.5 (91.0)	99.8 (97.3)
Total reflections	214,919	1,080,740
Unique reflections	55,264	50,739
*I*/σ	6.2 (1.8)	12 (1.8)
*R*_pim_^a^	8.4 (63)	3.9 (51.3)
CC half^b^	0.99 (0.58)	0.998 (0.708)
Multiplicity	3.9 (3.9)	21.3 (20.4)
Refinement		
Resolution range (Å)	38.86–2.5	39.88–1.94
No. of reflections	55,219	50,609
*R*_work_/*R*_free_^c^	20.55/25.08	22.53/25.58
No. of nonhydrogen atoms	11,824	12,085
Protein atoms	11,747	12,069
Ligand atoms	12	10
Waters	65	6
Ramachandran favored (%)	97.35	96.08
Ramachandran outliers (%)	0.07	0.20
Wilson *B*	46.12	89.3
Average *B* (Å^2^)	53.77	88.1
r.m.s. deviations		
Bond lengths (Å)	0.003	0.008
Bond angles (°)	0.565	0.884
PDB ID	36YL	9YQ9

Data were collected on beamlines 12-1 and 12-2 at the Stanford Synchrotron Radiation Lightsource.

Values in parentheses are for the highest resolution shells.

a *R*_pim_ = $\sum_{hkl}\sqrt{1/n-1}\left|I_{hkl}-{\left\langle I\right\rangle }_{hkl}\right|/\sum_{hkl}I_{hkl}$ where n is the multiplicity of related reflections.

b ${\text{CC}}_{1/2}$ = CC for random half sets (17).

c *R* = $\sum \left|F_{o}-\left|F_{c}\right|\right|/\sum \left|F_{o}\right|$ where $F_{o}$ = to the observed structure factors and $F_{c}$ = structure factors calculated from the model.

### BfCAPADH data collection and structure determination

Diffraction data for BfCAPADH were collected remotely using Blu-ICE (22) on beamline 12-1 at the SSRL. A total of 360° of data were collected at a wavelength of 0.9795 Å with 0.15° oscillations and 0.2 s exposures at a temperature of 100 K. The detector distance was 400 mm. Statistics for data collection and refinement data are listed in Table 4. The data were processed to 2.91 Å in XDS (23). The BfCAPADH structure was solved by molecular replacement using Phenix Phaser (24) with a 3.2 Å structure of BfCAPADH-Apo that was solved previously using chain A of PDB:8DEB as a model. The resulting log-likelihood gain was 7348.9 with a TFZ score of 53.0. The initial refinement following molecular replacement resulted in R_free_ = 31.65 and R_work_ = 26.56. Rounds of model building and refinement were completed in Coot and Phenix Refine (25). Waters were placed manually. The finished model was refined to R_free_ = 25.58 and R_work_ = 22.53. Ramachandran analysis was performed by MolProbity, showing 96.08% favored conformations with 0.2% outliers. The structure showed high atomic displacement parameters (Wilson B = 89). We solved three structures of this morphology at 3.3 Å, 3.15 Å and 2.91 Å to evaluate B-factors across different crystals and found them to be high in each case. The space group is large (a = 118 Å, b = 118 Å and c = 298 Å) with high solvent content allowing the loop movements that make this structure valuable while contributing to high B-factors. Residues with average B-factors >100 were removed even when apparent electron density was visible.

### Structural analysis

Structural comparisons were performed using the cealign function in PyMol. Structure figures were generated in PyMol (PyMol Molecular Graphics System, version 3.1, Schrodinger, LLC). The interface surface area was 987 Å^2^ for ClCAPADH and 1040 Å^2^ for BfCAPADH as calculated by PDBePISA (17).

### SAXS sample preparation and data analysis

ClCAPADH and BfCAPADH were purified as previously described with the following modification. A size exclusion chromatography purification step was added after nickel affinity chromatography as described previously (7). Samples for SAXS analysis were shipped to SSRL on dry ice, arrived frozen and were thawed immediately prior to use. Data were collected on beamline 4-2. Immediately before data collection, 32 μl samples were prepared in 0.2 ml 8× PCR strips in concentrations from 0.3 to 9.0 mg/ml (BfCAPADH) and 0.3 to 4.5 mg/ml (ClCAPADH) at 22 °C. Samples were diluted with 25 mM KPi pH 8 buffer (BfCAPADH) or 50 mM Tris pH 8 buffer (ClCAPADH) and contained 1 mM NADP^+^, or 700 μM NADPH, or no ligand. For each concentration series, scattering data were collected on a 64 μl sample of buffer before and after protein sample data collection to subtract the buffer contribution from final data sets. The PCR strips were transferred to the autosampler where each sample was injected into a 1.5 mm quartz capillary cell. Sample was exposed to x-rays for 10 s and 10 images were recorded. Scattering images were collected using a Rayonix MX225-HE detector at a sample-detector distance of 1.7 m and λ = 0.1128 nm. Scattering profiles were collected and observed using SAXSPipe. Data was analyzed using the ATSAS 3.0 Primus software. Guinier and p(r) function graphs were generated and used to determine the radius of gyration for each protein sample. Data were deposited in the Small Angle Scattering Biological Data Bank with accession numbers: SASDXU9 (ClCAPADH Apo), SASDXV9 (ClCAPADH-NADP^+^), SASDXW9 (ClCAPADH-NADPH), SASDXZ9 (BfCAPADH-NADPH), SASDXX9 (BfCAPADH-Apo), and SASDXY9 (BfCAPADH-NADP^+^).

### Multiple sequence alignment

Homologs of BfCAPADH with known substrate specificity were selected for sequence alignment analysis. ClCAPADH was selected as an agmatine utilizing homolog. Homologs from *B. abortus* (BaCASDH) and *A. tumefaciens* (AtCASDH) use putrescine as a substrate in the production of spermidine (8). *Vibrio cholerae* (VcCANSDH) uses diaminopropane in the production of norspermidine (4). The sequences of BfCAPADH (Uniprot ID I9VY56), ClCAPADH (Uniprot ID A7VSG1), BaCASDH (Uniprot ID Q2YPK8), AtCASDH (Uniprot ID A9CG66), and VcCANSDH (Uniprot ID Q9KRL3) were obtained by BLaST search using BfCAPADH as a query sequence in Uniprot. A structural alignment (Expresso) of BfCAPADH and its homologs was performed through the T-COFFEE Multiple Sequence Alignment Server. The resulting alignment was exported to ESPript 3.2 where the secondary structure of BfCAPADH was overlaid. Further annotation was added in Powerpoint (Fig. S11).

### Boltz-2 modeling

Protein-ligand docking models were generated using the Boltz-2 online modeling platform developed by Tamarind Bio. Amino acid sequences corresponding to the enzymes of interest were used as protein inputs, and the corresponding enzymatic substrates or products were provided as ligand inputs in SMILES format. The resulting models were visualized in PyMOL to evaluate the predicted ligand positioning within the active site regions of their corresponding enzymes. The highest-confidence predicted model generated by Boltz-2 was selected for further structural analysis.

## Data availability

Additional experimental data and data fitting may be found in the supplementary information. Further details can be shared upon a request made to the corresponding author. The ClCAPADH-Apo crystal structure and BfCAPADH-Apo crystal structure have been deposited under PDB accession numbers 36YL and 9YQ9. Small angle X-ray scattering data sets have been deposited under SASBDB accession numbers SASDXU9 (ClCAPADH Apo), SASDXV9 (ClCAPADH-NADP^+^), SASDXW9 (ClCAPADH-NADPH), SASDXZ9 (BfCAPADH-NADPH), SASDXX9 (BfCAPADH-Apo), and SASDXY9 (BfCAPADH-NADP^+^).

## Supporting information

This article contains supporting information.

## Conflict of interest

The authors declare that they have no conflicts of interest with the contents of this article.
