# Supplementary material for: Agmatine as the initiating substrate for aspartate semialdehyde-dependent bacterial spermidine biosynthesis
Source: J Biol Chem. 2026 Sep 17;302(11):113570. doi: 10.1016/j.jbc.2026.113570 (PMC13683441; doi:10.1016/j.jbc.2026.113570)
Supplement: Supplementary file 1 [file mmc1.docx]

**SUPPORTING INFORMATION**

**Agmatine as the initiating substrate for aspartate semialdehyde-dependent bacterial spermidine biosynthesis**

Johnette C. Ostlund, Lindsey A. Johnston, Makenzie A. Pardoe, Isabel M. Bustillos, Megan L. Bizzell, Savannah J. Jones, Danielle F. Lee, Jeffrey S. McFarlane^*^

**Table of Contents:**

1 – Table S1. LC-MS/MS assay of spermidine biosynthesis.

2 – Figure S1. Secondary plots of CAPADH steady-state kinetic data.

4 – Figure S2. *B. fragilis* stopped-flow assay of aminopropylagmatine (APA) production.

5 – Table S2. CAPADH thermal melt temperatures

5 – Figure S3. BfCAPADH thermal shifts

6 – Figure S4. NADPH Binding Assays by Stopped-Flow Spectrometry

6 – Figure S5. NADPH Binding in the presence of ASA

7 – Figure S6. NADPH Binding in the presence of putrescine

8 – Figure S7. NADPH Binding in the presence of agmatine

8 – Figure S8. NADPH/NADP+ Binding Competition Assays

9 – Table S3. Model Components

10 – Figure S9. CAPADH SAXS data

11 – Figure S10. 2.91 Å BfCAPADH-Apo Electron Density Maps

12 – Figure S11. Annotated CAPADH sequence alignment

**Table S1. LC-MS/MS assay of spermidine biosynthesis.** Product ion (446 *m/z*) peak areas for spermidine and ^13^C spermidine. DH – *B. fragilis* carboxyaminopropylagmatine dehydrogenase. DC – *B. fragilis* carboxyaminopropylagmatine decarboxylase.

**Figure S1. Secondary plots of CAPADH steady-state kinetic data**. Initial rates were determined by applying linear fits to primary plots of NAD(P)H oxidation at 340 nm. Secondary plots were fit with the Michaelis-Menten equation. Error bars represent the standard deviation of at least three trials. **A.** BfCAPADH (1 µM), ASA (aspartate semi-aldehyde – 1 mM), agmatine (1.25 mM) and NADPH varied from 0.1 to 50 µM. **B.** BfCAPADH (1 µM), ASA (1 mM), NADPH (150 µM), and agmatine varied from 78 µM to 5 mM. **C.** BfCAPADH (1 µM), agmatine (1.25 mM), NADPH (150 µM), and varied ASA from 19.5 µM – 1.25 mM. **D.** ClCAPADH (0.2 µM), ASA (1 mM), agmatine (1.25 mM) and NADPH varied from 0.1 to 25 µM. **E.** ClCAPADH (0.2 µM), ASA (1 mM), NADPH (150 µM), and agmatine varied from 1.2 µM to 2.5 mM. **F.** ClCAPADH (0.2 µM), agmatine (1.25 mM), NADPH (150 µM), and varied ASA from 39 µM – 1.25 mM.

**Figure S2. *Bacteroides fragilis* stopped-flow assay of aminopropylagmatine (APA) production.** Pyridoxal-5’-phosphate absorbance was monitored for 3.2 s from 300-550 nm using a TgK Scientific KinetaScan CCD with 1.6 ms scan integration times. All concentrations are the final concentration in the stopped-flow cell. BfCAPADC (25 µM) was rapidly mixed with CAPA (6.25 - 50 µM). Multiwavelength data were analyzed in Kintek Explorer by single value decomposition. Fits – dashed lines. Experimental data – gray circles. Orange – initial scan. Pink – final scan. Purple – intermediate scan. **A.** 12.5 µM CAPA. **B.** 25 µM CAPA. **C.** 50 µM CAPA. **D.** FitSpace analysis of global fitting to a two-step, irreversible model. **E.** Raw spectra for experiment in panel A. Every 10^th^ scan and every 3^rd^ data point are shown for clarity. **F.** Raw spectra. BfCAPADC (25 µM) mixed with 12.5 µM CSP. Every 10^th^ scan and every 3^rd^ data point are shown for clarity. Traces are nearly overlaid demonstrating little spectral change with CSP as a substrate.

**Table S2. CAPADH thermal melt temperatures.** ASA – asparate semialdehyde, CSP – carboxyspermidine.

**Figure S3. BfCAPADH thermal shifts.** BfCAPADH at 10 µM was combined with Sypro Orange and ligand at 10-fold excess over its *K_m_* value. Plots are the 1^st^ derivative of the melting curves. The apex of each peak represents the melting temperature (*T_m_*). Each curve is the average three different experiments with six technical replicates in each experiment.

**Figure S4.** **NADPH Binding Assays by Stopped-Flow Spectrometry.** The NADPH nicotinamide ring was excited at 340 nm and fluorescence emission was measured beyond a 360 nm cut-off filter. All concentrations are the final mixed concentration in the stopped-flow cell. For each experiment, BfCAPADH in one syringe was mixed against a 2-fold dilution series of NADPH in a second syringe. **A.** BfCAPADH (15 µM) mixed with NADPH (3.125-50 µM). **B.** BfCAPADH (25 µM) mixed with NADPH (10-40 µM). **C.** BfCAPADH (15 µM) mixed with NADPH (3.75-60 µM). **D.** BfCAPADH (15 µM) mixed with NADPH (0-36 µM). **E.** BfCAPADH (15 µM) mixed with NADPH (0-36 µM). **F.** Fitspace analysis to demonstrate determined rate constant boundaries. Data sets A-E were globally fit to model 1 (E = EN).

**Figure S5.** **NADPH Binding in the presence of ASA.** The NADPH nicotinamide ring was excited at 340 nm and fluorescence emission was measured beyond a 360 nm cut-off filter. All concentrations are the final mixed concentration in the stopped-flow cell. BfCAPADH at 15 µM was mixed against 30 µM NADPH combined with varied ASA from 3.75-60 µM in a second syringe.

**Figure S6.** **NADPH Binding in the presence of putrescine.** The NADPH nicotinamide ring was excited at 340 nm and fluorescence emission was measured beyond a 360 nm cut-off filter. All concentrations are the final mixed concentration in the stopped-flow cell. For each experiment, BfCAPADH in one syringe was mixed against a 2-fold dilution series of NADPH in a second syringe. **A.** BfCAPADH (15 µM) mixed with 30 µM NADPH and putrescine varied from 0.47 µM to 120 µM. Only the 0.47, 7.5 and 120 µM trials are shown for clarity. **B.** BfCAPADH (15 µM) mixed with 2 mM putrescine and NADPH varied from 0.47 – 30 µM. **C.** BfCAPADH (15 µM) mixed with 2 mM putrescine and NADPH varied from 0.47 – 30 µM. **D.** Fitspace analysis to demonstrate determined rate constant boundaries. Data sets B and C were globally fit to model 1 (E = EN).

**Figure S7.** **NADPH Binding in the presence of agmatine.** The NADPH nicotinamide ring was excited at 340 nm and fluorescence emission was measured beyond a 360 nm cut-off filter. All concentrations are the final mixed concentration in the stopped-flow cell. **A.** BfCAPADH at 15 µM mixed with 2 mM agmatine and NADPH varied from 0.47-7.5 µM. **B.** BfCAPADH at 15 µM mixed with 30 µM NADPH and agmatine varied from 3.75-60 µM and 1.25 mM. **C.** BfCAPADH at 15 µM mixed with 30 µM NADPH and agmatine varied from 0.47-120 µM. **D.** BfCAPADH at 15 µM mixed with 2 mM agmatine and NADPH varied from 0.47-30 µM. **E.** Fitspace analysis to demonstrate determined rate constant boundaries. Datasets were globally fit in Kintek Explorer to model 2 (EN=E=EA=EAN).

**Figure S8.** **NADPH/NADP+ Binding Competition Assays.** The NADPH nicotinamide ring was excited at 340 nm and fluorescence emission was measured beyond a 360 nm cut-off filter. All concentrations are the final mixed concentration in the stopped-flow cell. For each experiment, BfCAPADH in one syringe was mixed against NADPH and a 2-fold dilution series of NADP+ in a second syringe. **A.** BfCAPADH (15 µM) mixed with NADPH (30 µM) and NADP+ (3.75-60 µM). **B.** BfCAPADH (15 µM) mixed with NADPH (120 µM) and NADP+ (3.75-60 µM). The highest amplitude change in each trial corresponds to the lowest quantity of NADP+. **C.** Fitspace analysis to demonstrate determined rate constant boundaries. Datasets were globally fit to model 3 (EP=E=EN).

**Table S3. Model Components**

| Protein | ASU | Ordered Residues | Waters |
| --- | --- | --- | --- |
| ClCAPADH - Apo | 4 | Chain A: 2-85, 88-106, 118-170, 185-291, 302-399  Chain B: 1-179, 182-399  Chain C: 1-110, 118-179, 182-399  Chain D: 2-85, 89-106, 121-171, 191-291, 304-399 | 7  28  26  4 |
| BfCAPADH - Apo | 4 | Chain A: 1-168, 185-289, 301-397  Chain B: 1-168, 185-277, 280-289, 302-330, 338-397  Chain C: 1-108, 115-397  Chain D: 3-96, 100-108, 115-397 | 2  0  4  0 |

**Figure S9. CAPADH SAXS data**. Guinier and p(r) function plots are shown for each dataset. R_g_= radius of gyration (Å). D_max_= maximum diameter (Å). A-B. BfCAPADH Apo. C-D. ClCAPADH Apo. E-F. BfCAPADH NADP+. G-H. BfCAPADH NADPH. I-J. ClCAPDH NADP+. K-L. ClCAPADH NADPH.

**Figure S10. 2.91 Å BfCAPADH-Apo Electron Density Maps**. Gray mesh is 2*F_o_*-*F_c_* electron density map contoured at 1.5 $\sigma$. A. $\beta$7-α8 loop (purple). B. α11-$\beta$15 loop (yellow). Coloring matches Fig. 7.

**Figure S11. Annotated CAPADH sequence alignment**.
